# Protective Mechanism of Gandou Decoction in a Copper-Laden Hepatolenticular Degeneration Model: *In Vitro* Pharmacology and Cell Metabolomics

**DOI:** 10.3389/fphar.2022.848897

**Published:** 2022-03-23

**Authors:** Fengxia Yin, Mengnan Nian, Na Wang, Hongfei Wu, Huan Wu, Wenchen Zhao, Shijian Cao, Peng Wu, An Zhou

**Affiliations:** ^1^ The Experimental Research Center, Anhui University of Chinese Medicine, Hefei, China; ^2^ School of Pharmacy, Anhui University of Chinese Medicine, Hefei, China; ^3^ Anhui Province Key Laboratory of Research and Development of Chinese Medicine, Anhui Province Key Laboratory of Chinese Medicinal Formula, Hefei, China; ^4^ Department of Pharmaceutical Sciences, School of Pharmacy, University of Pittsburgh, Pittsburgh, PA, United States; ^5^ The First Affiliated Hospital of Anhui University of Chinese Medicine, Hefei, China

**Keywords:** gandou decoction, copper-laden HLD hepatocyte model, hepatolenticular degeneration, cell metabolomics, pharmacology

## Abstract

Gandou decoction (GDD) is a classic prescription for the treatment of hepatolenticular degeneration (HLD) in China; however, the liver-protecting mechanism of this prescription needs further evaluation. In the present study, we explored the protective mechanisms of GDD in a copper-laden HLD model using integrated pharmacology and cellular metabolomics *in vitro*. The results revealed that GDD could significantly promote copper excretion in copper-laden HLD model cells and improve the ultrastructural changes in hepatocytes. In addition, GDD could decrease the extent of lipid peroxidation, levels of reactive oxygen species, and the release rate of lactate dehydrogenase while increasing the activity of superoxide dismutase and the ratio of glutathione to oxidized glutathione in the copper-laden HLD model cells. On conducting statistical analysis of significant metabolic changes, 47 biomarkers and 30 related metabolic pathways were screened as pharmacological reactions induced by GDD in HLD model cells. d-glutamate and d-glutamine metabolic pathways showed the highest importance and significance among the 30 metabolic pathways, and the differential expression levels of the glutamine synthetase (GS) and the renal type and liver type GLS (GLS1 and GLS2) proteins were verified by Western blotting. Collectively, our data established the underlying mechanism of GDD therapy, such as the promotion of copper excretion and improvement in oxidative stress by regulating the expressions of GS, GLS1, and GLS2 protein to protect hepatocytes from injury.

## Graphical Abstract

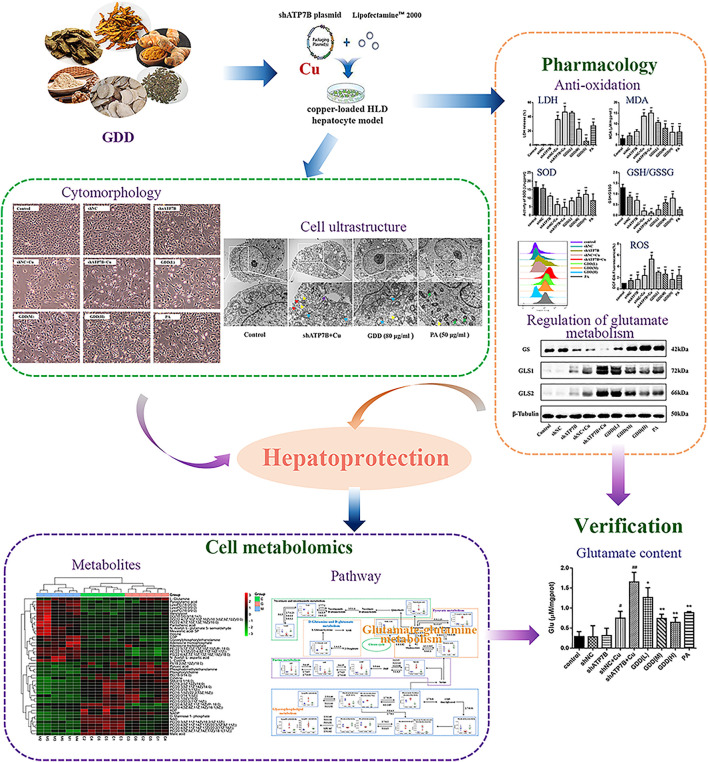

## 1 Introduction

Hepatolenticular degeneration (HLD), also known as Wilson's disease (WD), is a genetic copper overload disease caused by inactivating mutations in the copper transporter P-type ATPase gene (ATP7B) (Ala et al., 2007). Disease-causing mutations of ATP7B lead to impaired intracellular copper trafficking and consequent high copper accumulation in tissues, especially in the liver (Tanzi et al., 1993; Terada et al., 1998). Owing to the deposition of copper in the liver, the most typical disorders of HLD patients include hepatic diseases such as asymptomatic hepatomegaly, hepatic damage, liver fibrosis, cirrhosis, progressive systemic sclerosis, and liver failure (Bandmann et al., 2015). Like few other genetic and metabolic disorders, HLD can effectively be maintained with pharmacological agents. The most useful and direct clinical treatment for HLD is copper chelation therapy using copper-chelating agents, *e.g.*, D-penicillamine, trientine, tetrathiomolybdate, and dimercaptosuccinic acid, which can promote the elimination of copper. Unfortunately, copper-chelating agents usually have multiple toxic adverse effects, including immunologically induced lesions, renal toxicity, and poor compliance, which consequently result in failure of treatment (Hedera, 2017; Liu et al., 2017). Therefore, more effective and less toxic medications are desperately needed for the treatment of HLD.

Gandou decoction (GDD) is a classic compound prescription in traditional Chinese medicine (TCM) and is composed of six Chinese medicinal herbs: *Rheum palmatum* L. (Chinese name: Dahuang), *Coptis chinensis* Franch. (Chinese name: Huanglian), *Curcuma longa* L. (Chinese name: Jianghuang), *Lysimachia christinae* Hance (Chinese name: Jinqiancao), *Alisma orientale* (Sam.) Juzep. (Chinese name: Zexie), and *Panax notoginseng* (Burk.) F. H. Chen (Chinese name: Sanqi) in fixed proportions. In China, GDD has widely been used as an effective clinical therapeutic agent against HLD for decades. Previous clinical studies have demonstrated that GDD can not only significantly promote the excretion of copper and markedly improve the clinical manifestations of HLD patients but also exhibit significant liver protection (Xue et al., 2007; Wang et al., 2012; Xu et al., 2012). Given the great clinical therapeutic effects and high safety of GDD, it has been recommended for the clinical treatment of HLD patients as a basic prescription. In our previous study, we have revealed the chemical profile and the metabolites of GDD *in vitro* and *in vivo* based on ultra-performance liquid chromatography/quadrupole time-of-flight mass spectrometry (UPLC-Q-TOF-MS^E^). In total, 96 chemical constituents, including protostane triterpenoids, flavonoids, triterpenoid saponins, tannins, curcuminoids, and anthraquinones, have been identified in GDD (Xu et al., 2019). Based on these results, we further identified prototypes and metabolites of GDD in the serum, liver, and urine after oral administration to normal and copper-laden rats (an animal model of HLD) (Xu et al., 2020; Zhang et al., 2020). Meanwhile, we found that GDD could reduce copper accumulation (Cheng et al., 2018), regulate metabolic profile (Xu et al., 2021), and alleviate hepatic injury by inhibiting oxidative stress in HLD model rats (Wang et al., 2020). Taken together, GDD could effectively treat HLD with several benefits. However, the biological mechanism and significance for these effects were still not clear and needed further study.

Metabolomics focuses on subtle changes in metabolites (relative molecular mass less than 1,000) in complex biological systems (such as cells, tissues, or organisms) and is applied to investigate disease pathogenesis, evaluate the therapeutic effects, and discover potential biological mechanisms (Yang et al., 2019). Cell metabolomics, an important embranchment of metabolomics, is capable of directly revealing the pathological changes and pathogenesis of the disease at a metabolic level. Compared with the tissues or organisms, cell metabolomics has obvious advantages such as lower costs, better stability and reproducibility, fewer ethical issues, among others (Chrysanthopoulos et al., 2010; Cuperlović-Culf et al., 2010). In the past few years, cell metabolomics has been used for revealing the therapeutic mechanisms underlying the use of herbs alone or TCM formulae in diseases (Sun et al., 2018; Zhang et al., 2019; Ma et al., 2020). The major analytical techniques in metabolomics are nuclear magnetic resonance (NMR) spectroscopy, gas chromatography–mass spectrometry (GC-MS), capillary electrophoresis–mass spectrometry (CE-MS), and liquid chromatography–mass spectrometry (LC-MS). UPLC-Q-TOF/MS quickly became the preferred tool in cell metabolomics studies for its high resolution, high sensitivity, good reproducibility, and less sample requirement (Wu et al., 2020). The conventional reverse-phase (RP) column commonly used in UPLC-Q-TOF/MS mainly focuses on the middle and nonpolar metabolites. In contrast, the hydrophilic interaction liquid chromatography (HILIC) column is ideal for the analysis of polar metabolites. Therefore, a combination of RPLC and HILIC may be able to ensure maximum coverage in metabolomics (González-Ruiz et al., 2017).

Pieces of evidence from clinical and animal studies show that GDD has good therapeutic action against HLD. In this report, the rat liver-derived cell line BRL-3A was used to clarify the protective effect and metabolism mechanism of GDD. First, the ATP7B short-hairpin RNA (shRNA) plasmid transient transfection technique was used to damage BRL-3A cells, establishing an HLD hepatocyte injury model. The cytoprotective effect of GDD was then evaluated by detecting the changes in intracellular copper levels. Second, oxidative stress-related indicators, including superoxide dismutase (SOD), lipid peroxidation (MDA), glutathione (GSH) to oxidized glutathione (GSSH) level, and reactive oxygen species (ROS) level, were determined. Moreover, the ultrastructure of hepatocytes was observed under a transmission electron microscope (TEM). Third, the cellular untargeted metabolomics method based on UPLC-Q-TOF/MS was used to identify the biomarkers for exploring the potential mechanism of GDD in HLD at a cellular level. Finally, on this basis, enzymes in the critical metabolic pathways were verified by Western blotting.

## 2 Materials and Methods

### 2.1. Reagents and Chemicals

RPMI1640 was purchased from Corning (Manassas, United States). Fetal bovine serum (FBS) was purchased from Hyclone (Logan, United States). Trypsin digestion solution was purchased from Beyotime Biotechnology (Shanghai, China). Penicillin–streptomycin solution was obtained from Service Biotechnology (Wuhan, China). Furthermore, 3-(4,5-dimethyl-thiazol-2-yl)-2,5-diphenyl-tetrazolium bromide (MTT) was purchased from BioFroxx (Einhausen, Germany). shRNA plasmid vector kit was purchased from GenePharma Co. Ltd. (Shanghai, China). Lipofiter (batch number: 20201202) was purchased from Shanghai Hanheng Biotechnology Co., Ltd. Phosphate buffer saline (PBS) was purchased from Suobao Biotechnology Co., Ltd. (Shanghai, China). Lipid oxidation (MDA) detection kit, total superoxide dismutase (T-SOD) activity test kit, reactive oxygen species (ROS) detection kit, glutathione (GSH) and oxidized glutathione (GSSH) detection kit, Bradford protein concentration determination kit, and lactate dehydrogenase (LDH) cytotoxicity test kit were all purchased from Beyotime Biotechnology Co., Ltd. (Shanghai, China). Glutamate (Glu) content detection kits were obtained from Solarbio Science and Technology Co., Ltd. (Beijing, China). A rabbit monoclonal antibody, GLS1, was purchased from Abcam (Cambridge, United Kingdom). Mouse anti-*β*-tubulin monoclonal antibody, HRP-labeled goat anti-mouse lgG, and HRP-labeled goat anti-rabbit lgG were all purchased from Zhongshan Jinqiao Biological Technology Co., Ltd. (Beijing, China). ECL hypersensitive photoluminescence solution was purchased from Affinity Biologicals (Cincinnati, United States). The six herbs of *R. palmatum* L. (Batch number: 20180106), *C. chinensis* Franch. (Batch number: 20180101), *C. longa* L. (Batch number: 20180120), *L. christinae* Hance (Batch number: 20180113), *A. orientale* (Sam.) Juzep. (Batch number: 20180107), and *P. notoginseng* (Burk.) F. H. Chen (Batch number: 20180109) were purchased from Beijing Tongrentang Pharmacy Co., Ltd. (Hefei, China) and identified by Professor Rongchun Han, an expert from Traditional Chinese Pharmacology of the Anhui University of Chinese Medicine. All herbs had been deposited at the Herbarium of the Anhui University of Chinese Medicine, Hefei, China (Herbarium code: ACM, voucher numbers: 18,056, 18031,18,059, 18,070, 18,052, and 18,067). The details of drug materials are provided in Supplementary Table S1.

### 2.2 Preparation of Gandou Decoction

According to the prescription, a mixture of *R. palmatum* L. (10 g), *C. chinensis* Franch. (10 g), *C. longa* L. (10 g), *L. christinae* Hance (12 g), *A. orientale* (Sam.) Juzep. (12 g), and *P. notoginseng* (Burk.) F. H. Chen (1.5 g) was crushed using a high-speed pulverizer. After mixing, eightfold in water was added to extract and reflux for an hour each; this was repeated two times. The filtrate was strained and combined, then evaporated to a certain volume, and vacuum freeze-dried into a powder (15.3% yield). The quality of GDD was evaluated by measuring the contents of curcumin (16.45 mg/g), chrysophanol (6.69 mg/g), rhein (7.15 mg/g), physcion (4.41 mg/g), emodin (3.63 mg/g), aloe-emodin (3.16 mg/g), quercetin (3.44 mg/g), and kaempferide (1.99 mg/g) *via* high-performance liquid chromatography method, according to our previous research (Wang et al., 2020).

### 2.3 Cell Culture

The BRL-3A cells (ATCC: CRL-1442) were acquired from the Shanghai cell bank of the Chinese Academy of Sciences. The cells were cultured in RPMI-1640 medium with 10% FBS and 1% penicillin–streptomycin at 37°C in a 5% CO_2_ atmosphere. Passaging was conducted every 2–3 days, and the cells in the logarithmic growth phase were selected for the following experiments.

### 2.4 Establishment of Copper-Laden Hepatolenticular Degeneration Hepatocyte Injury Model Induced by CuSO_4_

In this study, screening was performed by 4′,6-diamidino-2-phenylindole (DAPI) staining method, real-time polymerase chain reaction (qPCR), and Western blotting (Supplementary Information S1.1). The method of RNA interference was used to establish the HLD hepatocyte model. The plasmid transfection group of shATP7B-4 with the strongest gene interference effect was chosen for the study of the copper-laden HLD hepatocyte injury model (Supplementary Information 2.1).

Subsequently, the copper-laden HLD hepatocyte injury model was established by CuSO_4_ induction, which simulates the copper-overload phenomenon of HLD patients. Approximately 5 × 10^3^/100 μl BRL-3A cells were seeded in a 96-well plate. After being transfected with shATP7B plasmid for 48 h, CuSO_4_ (0, 100, 200, 300, 400, 500, and 600 μM) was added with 5% FBS, and the cells were incubated for 12, 24, 36, and 48 h. At the same time, the control group and the shNC group were used as the controls, and six repeating holes were set up in each concentration. The cells in each group were incubated with 10-μl MTT solution (5 mg/ml) for 4 h at 37°C. The supernatant was discarded, and 150-μl DMSO was added to dissolve formazan. The optical density (OD) values were measured at 490 nm, and the cell viability (CV) (%) was calculated from the mean OD values of the six wells according to the following formula: CV (%) = (OD _Test_ – OD _Blank control_)/(OD _Control_ – OD _Blank control_) × 100%.

### 2.5 Evaluation of the Protective Effect of Gandou Decoction on Copper-Laden Hepatolenticular Degeneration Hepatocyte

Approximately 5 × 10^3^ HLD hepatocytes/100-μl solution were seeded in a 96-well plate, and various concentrations on GDD (10, 20, 40, 80, 160, and 320 μg/ml) were added. The follow-up operation was the same as that mentioned in Section 2.4. Subsequently, the CV (%) was calculated, viability above 90% indicated a safe concentration.

After administering the low, medium, and high doses of GDD (20, 40, and 80 μg/ml) and a dose of penicillamine (PA) (50 μg/ml), the experiment was divided into nine groups: control group, shNC group, shATP7B group, shNC + Cu group, shATP7B + Cu group, low, medium, and high GDD group, and PA group. The CV (%) was then calculated, as mentioned before.

### 2.6 Study on the Effect of Copper Excretion on Gandou Decoction in Copper-Laden Hepatolenticular Degeneration Hepatocytes

#### 2.6.1 Observation of Cu^2+^ Fluorescence Intensity in Cells

Under a fluorescence microscope (Olympus CKX41, Olympus Crop., Japan), the fluorescence intensity of intracellular copper (Duan et al., 2019), which indicated the intracellular copper level, was observed using the PP-Cu fluorescence probe. The cells were divided into nine groups following the same steps mentioned in Section 2.5. The culture medium was absorbed, and then, PP-Cu fluorescent probe (0.5 μM) was added to each well. After incubation for 1 h, the supernatant was discarded. This was followed by washing two times with PBS, and the fluorescence intensity of intracellular Cu^2+^ was observed under a fluorescence microscope using the green light excitation channel. Three visual fields were randomly selected for each group.

#### 2.6.2 Measurement of Cu^2+^ Content in Copper-Laden Hepatolenticular Degeneration Hepatocytes

Cells were collected and washed with PBS twice. Then, the cells were resuspended in PBS, crushed by ultrasonic waves in an ice bath, and centrifuged (4°C, 12,000 rpm) for 20 min. The supernatant (cytoplasm) of the cells was then obtained. The intracellular Cu^2+^ content was measured by Bradford protein quantification and atomic absorption spectrometry (AAS) (Thermo Corp., United States) (Liu et al., 2016). The ratio between copper and protein (nanogram per milligram) in each group was calculated as actual intracellular Cu^2+^, and the experiment was repeated three times.

### 2.7 Protective Effect of Gandou Decoction on Copper-Laden Hepatolenticular Degeneration Hepatocytes

#### 2.7.1 Morphological Observation

An inverted microscope was used to observe the morphological changes in nine groups of cells. After transfection for 48 h, different concentrations (20, 40, and 80 μg/ml) of GDD and PA were administered to cells in the corresponding groups and maintained for 6 h; the solution was then incubated in CuSO_4_ (300 μM) for 24 h. The cell morphology in each group was observed under an inverted microscope and photographed.

#### 2.7.2 Observation of Cell Ultrastructure

The cells were digested with 0.25% trypsin and centrifuged (4°C, 1,000 rpm) for 3 min to make a single-cell suspension. The suspension was stained with uranyl acetate and led citrate after fixation, dehydration, embedding, solidification, and sectioning, and then observed and photographed under a TEM.

#### 2.7.3 Measurement of Lipid Peroxidation, Total Superoxide Dismutase, Reactive Oxygen Species, Glutathione and Oxidized Glutathione, and Lactate Dehydrogenase

Ultrasonic pulverization was performed, followed by centrifugation for 10 min. Subsequently, the cell supernatant was collected in each group, and the levels of MAD and GSH/GSSH, as well as the activities of SOD, and the release of LDH were determined according to the instructions of the respective kits. In addition, the ROS content of the cells was determined using a fluorescence probe. Cells in each group were incubated with the dichlorodihydrofluorescein (DCFH-DA) probe (10 μM) for 20 min at 37°C, blending every 3–5 min. The cells were washed with serum-free medium three times, and the fluorescence intensity was detected by flow cytometry (excitation wavelength: 488 nm; emission wavelength: 525 nm).

### 2.8 Metabolite Extraction

The cells in the shNC group, shATP7B + Cu group, and (high-dose) GDD group were selected for the metabolomic study. Liquid–liquid extraction (Ivanisevic et al., 2013; Cuykx et al., 2017) was performed to extract metabolites from cells in each group. The extractant of methanol–ethyl acetate–water (2:2:1) was used to extract intracellular metabolites in each group. The cell Petri dish in each group was taken out and rinsed once with PBS (3 ml). Following this, methanol/water (2:1; 800 μl) was added, and the solution was frozen and thawed quickly three times (10 min at 25°C for 1 min under liquid nitrogen). The cells were then crushed using an ultrasonic cell disrupter (instrument parameters: working 5 s, interval 5 s, power 30%, working times: 4). Subsequently, 400-μl ethyl acetate was added, and the mixture was whirled for 30 s (repeated six times) after incubating in a cold-water bath for 5 min. It was then centrifuged at 13,000 g for 30 min at 4°C; the supernatant was extracted into a 1.5-ml EP tube and incubated at −80°C for 6–8 h. After delamination, two parts of the liquid were transferred into a new 1.5-ml EP tube (after layered, the top layer was used for RPLC-Q-TOF/MS analysis, and the bottom layer was used for HILIC-Q-TOF/MS analysis). The liquid was then blown dry with nitrogen and redissolved with 200 μl of the mobile phase. This was followed by centrifugation at 13,000×*g*/min under 4°C; the supernatant (100 μl) was placed in an injection vial. Meanwhile, quality control (QC) samples were prepared by mixing aliquots of 20 μl of shNC, shATP7B + Cu, and (high-dose) GDD group samples as described earlier.

### 2.9 Cellular Metabolomics Experiments

The cellular metabolomics study was conducted using the UPLC system (ACQUITY, WATERS, Milford, United States) coupled with a Xevo G2-XS Q-TOF/MS detector (ACQUITY, WATERS, United States). All samples were evaluated on an ACQUITY UPLC@HILIC column (2.1 mm × 100 mm, 1.7 μm) or an ACQUITY UPLC@BEH C_18_ column (2.1 mm × 100 mm, 1.7 μm). These two columns have used the separations under the following condition: column temperature, 38°C; sample room temperature, 4°C; flow rate, 0.2 ml/min, and injection volume, 1 μl. The mobile phase of the C_18_ column separation system consists of 0.1% formic acid aqueous solution (A) and 0.1% formic acid–acetonitrile (B), and the elution gradient of it was as follows: 5–12% A for 0–2.5 min, 12% A for 2.5–5 min, 12–32% A for 5–10 min, 32–29% for 10–11 min, 29–5% for 11–12 min, and 5% A for 12–15 min. For HILIC column separation, the mobile phase A was water and B was acetonitrile/isopropanol (5:2); both solvents contained 5-mM ammonium acetate and 0.1% formic acid. The elution gradient was set as follows: 0–2 min, 35–82% B; 2–9 min, 82–90% B; 9–14 min, 90–90% B; 14–15 min, 90–35% B; and 15–17 min, 35% B. QC samples were randomly distributed in the measured sample sequence.

In the Q-TOF/MS analysis part, optimal conditions for mass spectrometry were as follows: electrospray ionization (ESI) was used to produce ions; the temperatures of ion source were 120°C (+)/110°C (−), and the gas temperature was 350°C; the capillary voltage was 2.5kV/−2.0 kV; the gas flow rate was 600 L/h; the low collision energy was 6 V, and the high collision energy was 20–40 V; the mass range was 50–1200 Da. In the process of data acquisition, 200 ng/ml leucine enkephalin was used for real-time correction in the positive model (m/z 556.2771) and negative model (m/z 554.2615).

In addition, the QC sample was used for methodological investigations; it was injected six times continuously according to the experimental conditions mentioned earlier to investigate the precision of the instrument. Meanwhile, the QC sample was inserted into the injection sequence; that is, one QC sample was inserted between every four samples to investigate the stability of the sample (within 24 h after sample treatment). One ion peak was randomly selected in each column and each ion mode (Xu et al., 2021), and the relative standard deviation (RSD) of the ion peak area and retention time were calculated.

### 2.10 Data Processing and Multivariate Statistical Analyses

All experiments were repeated three times, and results are expressed as mean ± standard deviation. One-way analysis of variance (ANOVA) was performed by statistical inference using SPSS17.0 statistical software. For homogeneity of variance, the least significant difference (LSD) test was performed for multiple comparisons between groups, whereas the uneven variance was evaluated using Dunnett’s T3 test. A difference of *p* < 0.05 or *p* < 0.01 was considered statistically significant.

For metabolomics analysis, cell markers were studied using the Pregenic QI V 2.0 software and EZinfo 3.0 software, and the original data of UPLC-Q-TOF/MS were imported into the QI software for data processing (including baseline filtering, peak recognition, correction of retention time, and normalization). The filtered datasets were imported into EZinfo 3.0 software for multivariate statistical analysis. This software included unsupervised principal component analysis (PCA), which compared the differences among the shNC group, shATP7B + Cu group, and GDD group; it also observed the stability of the whole analysis process and supervised orthogonal partial least square discriminant analysis (OPLS-DA), which was used to screen metabolites with large metabolic differences among groups. The OPLS-DA model was verified by ANOVA of cross-validated residuals (CV-ANOVA). Differential variables are screened according to variable weight value (VIP) > 1.0.

### 2.11 Construction and Analysis of Metabolites and Metabolic Pathway

Accurate m/z obtained by multivariate statistical analysis was matched with the metabolites in the online database: Human Metabolomics database (HMDB) (which satisfies ±10 PPM). After evaluating the retention time and excimer ion information under low collision energy of mass spectrometry data, the ion fragments with high collision energy were compared with the HMDB (http://www.hmdb.ca/), which helped obtain potential metabolite biomarkers. Subsequently, normalized peak areas of potential metabolite biomarkers in each group were used to represent the relative content of corresponding metabolites in each group, and the differences in metabolites in each group were verified by one-way ANOVA. Finally, data information was imported into MetaboAnalyst 5.0 software, and the metabolic pathway was analyzed combined with the Kyoto Encyclopedia of Genes and Genomes database (http://www.kegg.jp/).

### 2.12 Western Blotting

The cells in each group were treated according to the method mentioned in Section 2.7.3. According to the instructions mentioned in the glutamate content detection kit, the level of glutamate in the cells was detected. Protein content was determined using the BCA protein detection kit. The loading buffer was added in equal amounts to the proteins. After sodium dodecyl sulfate (SDS)-polyacrylamide gel electrophoresis (PAGE), transfer membrane and sealing, the membrane and the antibody (GS: 1:1,000, GLS1: 1:500, and GLS2: 1:1,000) were incubated overnight at 4°C. Subsequently, goat anti-rabbit and goat anti-mouse antibodies were added at an appropriate dilution (diluted ratio: 1:15,000). Grayscale scanning and quantitative analysis were conducted using the Alphaview software.

## 3 Results

### 3.1 Effects of CuSO_4_ on Viability of shATP7B-Transfected Cells

**FIGURE 1:**
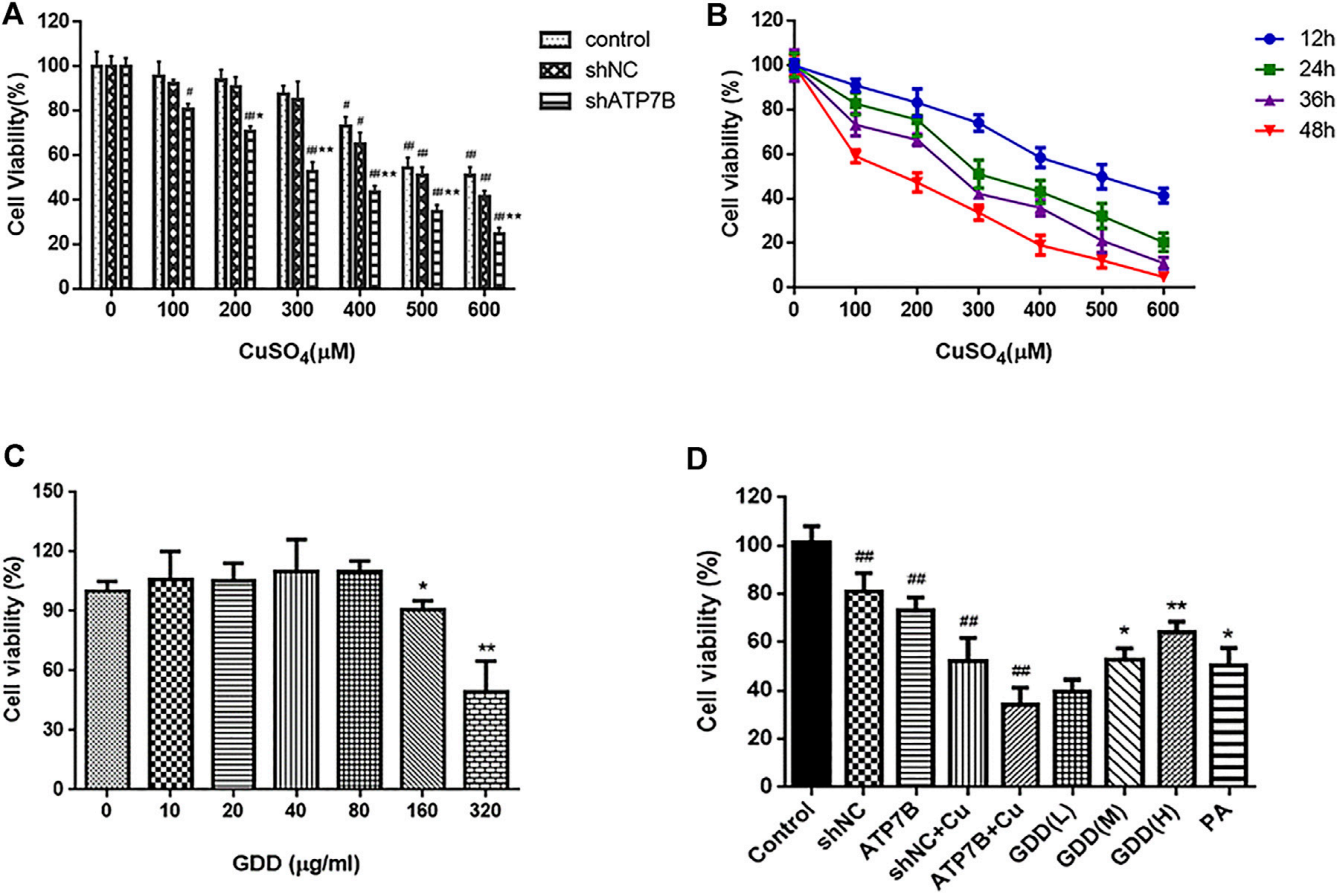
**(A)** Cell viability in control, shNC, and shATP7B transfected after 24-h incubation with different concentrations CuSO_4_ ( $\bar{x}\pm s,n=6$ ). ^#^ *p* < 0.05, ^##^ *p* < 0.01 compared with corresponding blank group without CuSO_4_ treatment; ^*^ *p* < 0.05, ^**^ *p* < 0.01 compared between shNC and shATP7B groups at same copper concentration; **(B)** dose and time-effect curves of shATP7B transfected cell viability after incubation with CuSO_4_ ( $\bar{x}\pm s,n=6$ ); **(C)** cell viability after 24 h treated by GDD ranging from 0 to 320 μg/ml (0, 10, 20, 40, 80, 160, and 320 μg/ml) ( $\bar{x}\pm s,n=6$ ). ^*^ *p* < 0.05, ^**^ *p* < 0.01 compared with control group; **(D)** cell viability in copper-laden HLD hepatocytes before and after GDD treatment ( $\bar{x}\pm s,n=6$ ). ^*^ *p* < 0.05, ^**^ *p* < 0.01 compared with shATP7B + Cu group.

As shown in Figure 1A, results showed that the cell survival rate in the shATP7B group was lower than that in other groups. Subsequently, shATP7B-transfected cells were incubated with CuSO_4_ solution ranging in concentration from 0 to 600 μM (0, 100, 200, 300, 400, 500, and 600 μM) for 12, 24, 36, and 48 h, and their MTT assays (Figure 1B) indicated that with an increase in copper concentration (the range of 100–600 μM CuSO_4_) and incubation time, the cell survival rate showed a certain dose- and time-dependent decrease. The IC_50_ value for CuSO_4_ was estimated to be 300 μM at 24 h, which was selected as an optimum modeling condition of copper-laden HLD hepatocytes.

### 3.2 Effect of Gandou Decoction on the Viability of Copper-Laden Hepatolenticular Degeneration Hepatocytes

The results of the MTT assay showed (Figure 1C) that GDD had no toxicity toward HLD hepatocytes in concentrations ranging from 10 to 80 μg/ml, which was considered a safe concentration range. Accordingly, concentrations of 20, 40, and 80 μg/ml GDD were selected for the low-, medium-, and high-dose groups, respectively, in the follow-up study. Similarly, the optimal dose of PA was determined to be 50 μg/ml.

The results for the HLD hepatocyte model experiments are shown in Figure 1D. The cell viabilities in the shNC group, shATP7B group, shNC + Cu group, and shATP7B+ Cu group were 81.20 ± 7.47%, 52.12 ± 9.76%, 73.08 ± 5.62%, and 34.19 ± 7.14% respectively. Compared with the shATP7B + Cu group, the groups with medium- and high-dose GDD treatment and penicillamine pretreatment showed an increased cell survival rate to varying degrees, and the cell viabilities in these groups were 53.00 ± 5.44%, 64.21 ± 4.40%, and 50.42 ± 7.19%, respectively. These results show that GDD has a strong protective effect on injured copper-laden HLD hepatocytes.

### 3.3 Excretion of Copper Promoted by Gandou Decoction in Copper-Laden Hepatolenticular Degeneration Hepatocytes

#### 3.3.1 Fluorescence Intensity of Cu^2+^ in Copper-Laden Hepatolenticular Degeneration Hepatocytes Affected by Gandou Decoction

**FIGURE 2:**
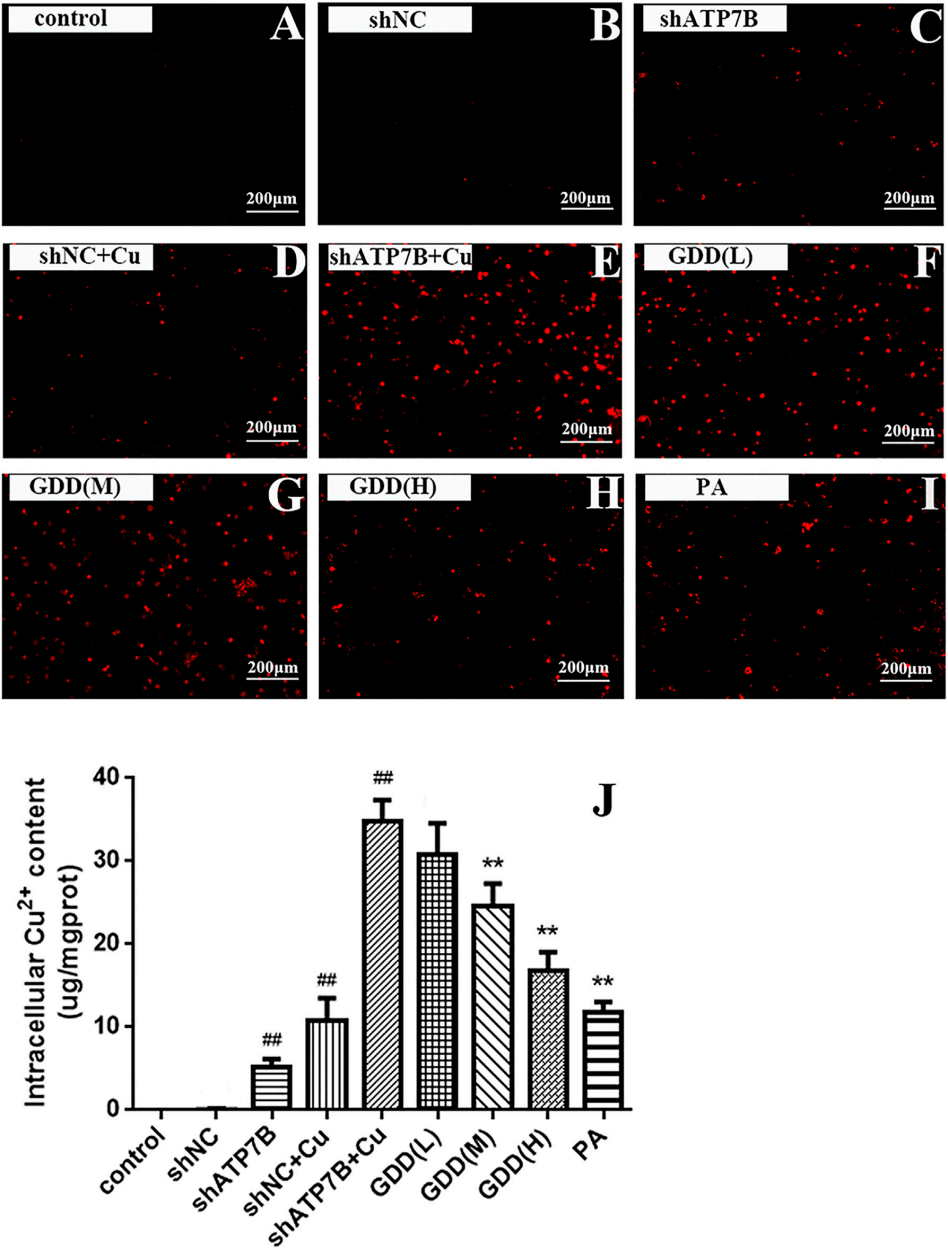
**(A–I)** Cu^2+^ fluorescence intensity of copper-laden HLD hepatocytes treated with GDD by fluorescence microscope (magnification = ×100); **(J)** effect of GDD on content of Cu^2+^ in copper-laden HLD hepatocytes ( $\bar{x}\pm s,n=6$ ). ^#^ *p* < 0.05, ^##^ *p* < 0.01 compared with control group; ^*^ *p* < 0.05, ^**^ *p* < 0.01 compared with shATP7B + Cu group.

As shown in Figures 2A–I, there was almost no red fluorescence in the cells in the control group, implying the little content of copper in these cells. After incubation with CuSO_4_, the weak red fluorescence in the shNC and shATP7B groups significantly increased, indicating an increase in intracellular Cu^2+^ content. Compared with the shNC + Cu group, the shATP7B + Cu group showed sparsely stained cells and stronger intensity of red fluorescence. However, after intervention with low, medium, and high doses of GDD and PA in the shATP7B + Cu group, the fluorescence intensity of copper decreased. The number of stained cells increased, especially in cells treated with high doses of GDD and PA. These results show that GDD could effectively inhibit the accumulation of copper and reduce its level in copper-laden HLD hepatocytes.

#### 3.3.2 Effect of Gandou Decoction on Cu^2+^ Content of Copper-Laden Hepatolenticular Degeneration Hepatocytes

The results of AAS determination showed that GDD has an effect on expelling copper. As shown in Figure 2J, the content of Cu^2+^ in the control group, shNC group, and shATP7B group was low, but that in the shATP7B + Cu group was significantly increased (*p* < 0.01), which indicated the accumulation of Cu^2+^ in copper-laden HLD hepatocytes. The copper content in cells treated with GDD and PA, especially high-dose GDD, was significantly lower than that in the shATP7B + Cu group (*p* < 0.05, *p* < 0.01).

### 3.4 Protective Effect of Gandou Decoction on Copper-Laden Hepatolenticular Degeneration Hepatocytes

**FIGURE 3:**
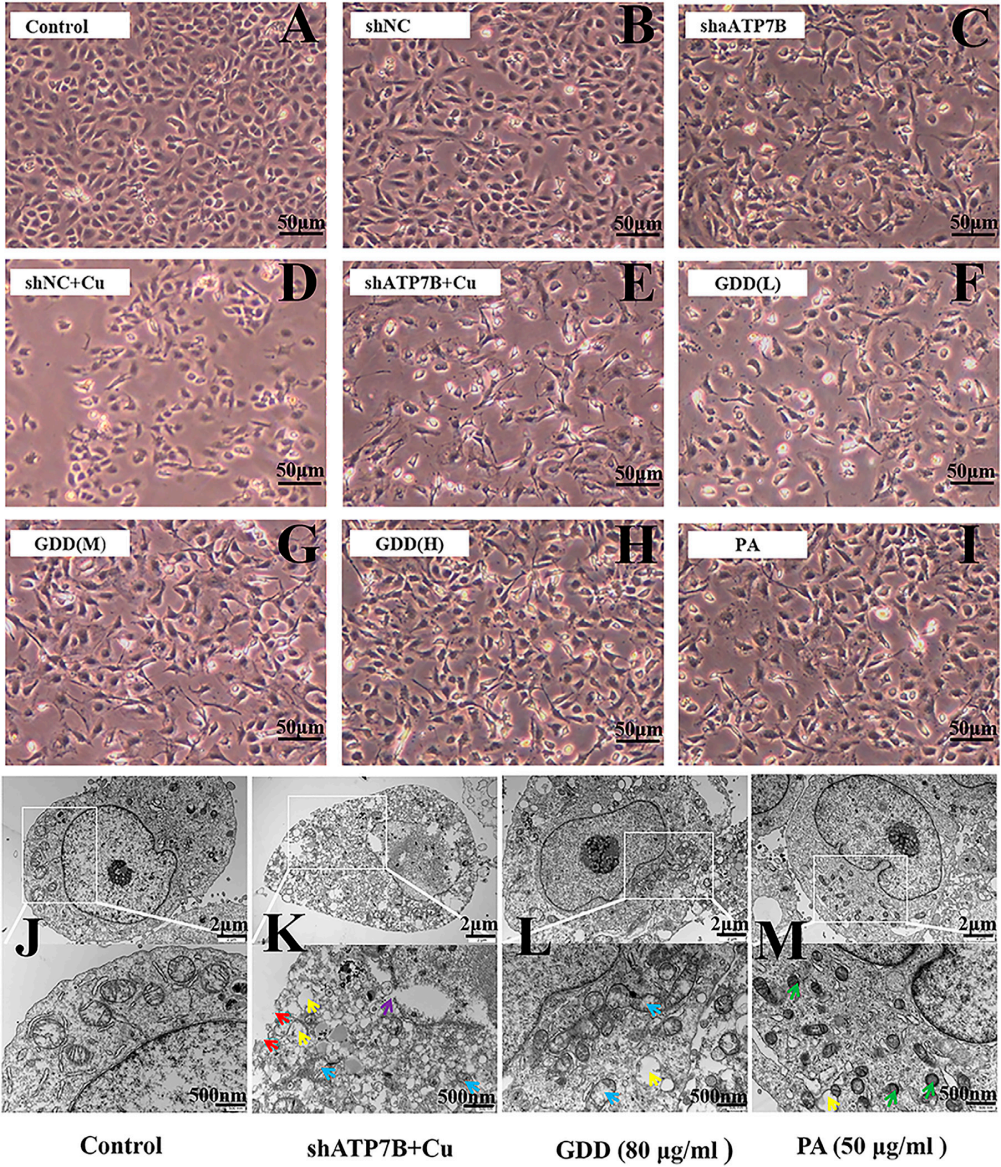
**(A–I)** Morphological changes of copper-laden HLD hepatocytes treated with GDD by inverted microscope (×200); **(J–M)** ultrastructure of copper-laden HLD hepatocytes observed by transmission electron microscope (×10,000 or ×25,000) (red arrow: cell membrane rupture; yellow arrow: mitochondrial vacuolation; purple arrow: nuclear membrane rupture; blue arrow: mitochondrial cristal rupture; green arrow: mitochondrial shrinkage).

Inverted microscope observation indicated that GDD could reduce injury in copper-laden HLD hepatocytes in different degrees. As shown in Figure 3A, in the control group, the cells showed a uniform flat or polygonal shape, adherent growth, complete cell membrane, and high density. After shATP7B transfection, the cell structure deteriorated, the cell membrane was sunken, and some cells were divided into fragments. Further through modeling by CuSO_4_, the number of cells significantly decreased, the number of suspended cells increased, cell membrane ruptured, cells shrunk, and cell damage intensified, indicating that CuSO_4_ can significantly inhibit cell growth (Figures 3B–E). However, after intervention with different concentrations of GDD and PA (Figures 3F–I), the cell status and degree of death improved. The number of viable cells increased compared with that in the shATP7B + Cu group.

Further evaluation of the changes of cell ultrastructure showed intact cell shape, round nucleus, complete and smooth nuclear membrane, various organelles in the cytoplasm, and lots of mitochondria in the control group (Figure 3J–M); even mitochondrial crista could be clearly seen. Compared with the control group, the shATP7B + Cu group showed a ruptured cell and nuclear membrane, disappeared nucleolus, decreased mitochondria number, blurred ridgeline of mitochondria, and obvious vacuolation. The structure of hepatocytes in GDD and PA groups was normal, with improvement in the blurred cristae and vacuolization of mitochondria.

### 3.5 Measurement of Biochemical Indices in Copper-Laden Hepatolenticular Degeneration Hepatocytes

**FIGURE 4:**
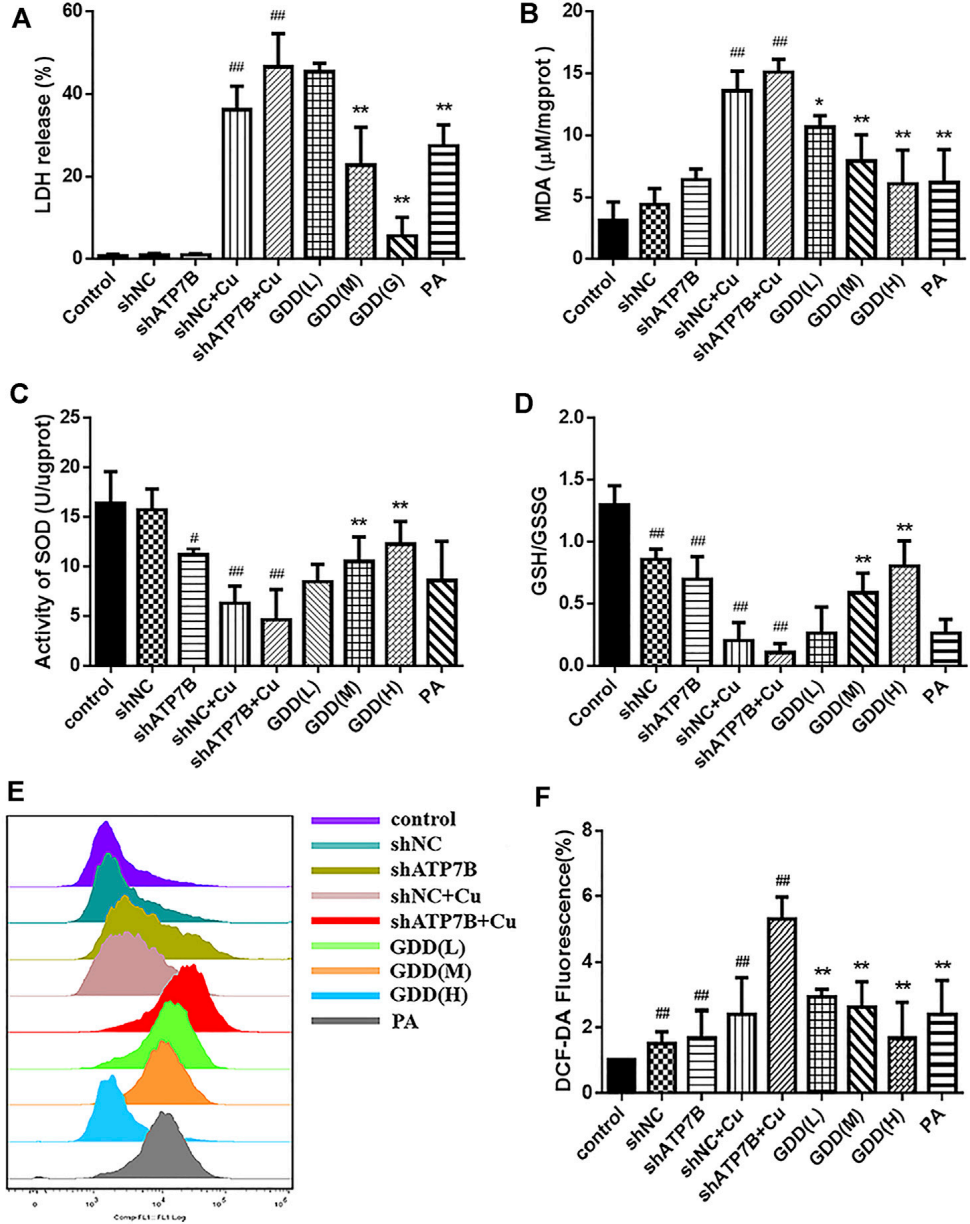
Effects of MDA **(A)**, LDH release **(B)**, SOD **(C)**, GSH/GSSG **(D)**, and ROS **(E, F)** levels in copper-laden HLD hepatocytes ( $\bar{x}\pm s,n=3$ ). ^#^ *p* < 0.05, ^##^ *p* < 0.01 compared with control group; ^*^ *p* < 0.05, ^**^ *p* < 0.01 compared with shATP7B + Cu group.

The results showed that the level of MDA in the shATP7B + Cu group was significantly higher than that in the control group (Figure 4B, *p* < 0.05), and intracellular MDA levels were effectively decreased after treatment with different doses of GDD and PA (*p* < 0.05). LDH is a relatively stable enzyme released outside the cell because of the destruction of the cell membrane structure, and it was regarded as an essential indicator of cell membrane integrity status. Compared with the control group (Figure 4A), the shATP7B + Cu group showed significantly increased LDH release. However, medium and high doses of GDD and PA could effectively reduce LDH release in HLD hepatocytes (*p* < 0.01). SOD, as the central defensive antioxidant enzyme in cells, directly participates in the decomposition of ROS in the body, reducing hepatocyte injury. The results of SOD detection showed that SOD activity in the shATP7B + Cu group was significantly lower than that in the control group (Figure 4C, *p* < 0.01). In contrast, the decrease in SOD activity was attenuated by medium- and high-dose GDD treatment (*p* < 0.01). As an antioxidant molecule, GSH plays an important role in intracellular redox balance, which is an important indicator of intracellular homeostasis. The results (Figure 4D) showed that after incubation with 300-μM CuSO_4_, GSH/GSSG levels in the shNC + Cu group and shATP7B + Cu group were only one-sixth and one-twelfth of the control group, respectively, indicating that the GSH in cells was almost completely suppressed. However, the medium- and high-dose GDD groups significantly inhibited the decrease in GSH/GSSG levels (*p* < 0.01). In addition, a DCFH-DA fluorescence probe was used to determine the content of ROS in cells. The results suggested that the fluorescence intensity in HLD hepatocytes significantly increased after CuSO_4_ induction for 24 h, indicating that CuSO_4_ promoted the release of ROS in cells (*p* < 0.01), and the fluorescence intensity-dependent reduction of DCF was caused by the pre-protection of the low, medium, and high doses of GDD. This indicated that GDD could reduce intracellular ROS. These results suggest that GDD has a significant protective effect against abnormal changes in copper-laden HLD hepatocytes (Figures 4E,F).

### 3.6 Multivariate Statistical Analysis

Combining the HILIC column and C_18_ column results, the total ion chromatograms (TICs) in the shNC group, shATP7B + Cu group, and GDD group were obtained with the full scan mode of dual ESI *via* UPLC-Q-TOF/MS analysis. The high reproducibility of the QC data demonstrated that the instrument is stable and the metabolomics analysis method is reliable (Supplementary Information S2.2). As shown in Supplementary Figure S2 and Supplementary Figure S3, there are differences in the metabolic profiles of samples between different groups in the positive and negative ion modes.

**FIGURE 5:**
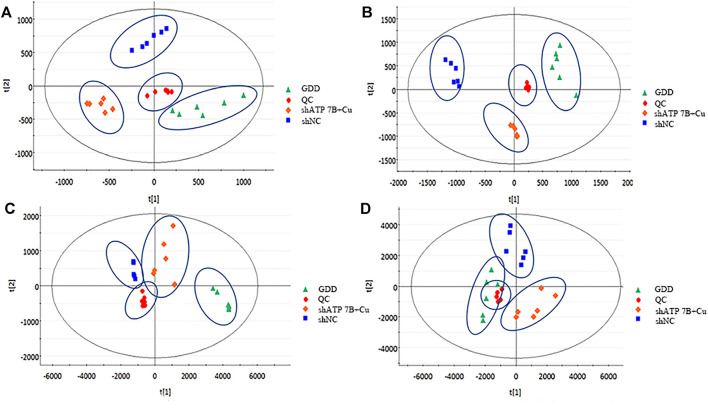
PCA score diagram of cell samples in negative **(A, C)** and positive **(B, D)** ESI modes. **(A, B)** HILIC-Q-TOF/MS [**(A)**: NEG-R^2^X = 83%, Q^2^ = 53%; **(B)**: POS- R^2^X = 97%, Q^2^ = 82%], **(C, D)** RPLC-Q-TOF/MS [**(C)**: NEG-R^2^X = 99%, Q^2^ = 97%; **(D)**: POS- R^2^X = 98%, Q^2^ = 94%]. Each point represents a cell sample.

The data were analyzed by the pattern recognition method to reveal the differences among the three groups under positive and negative ion modes. Given that in unsupervised multivariate analysis, PCA can truly reflect the clustering of samples, on processing data by PCA, it was found that there were differences among the shNC group, shATP7B + Cu group, and GDD group under negative and positive modes (Figure 5).

**FIGURE 6:**
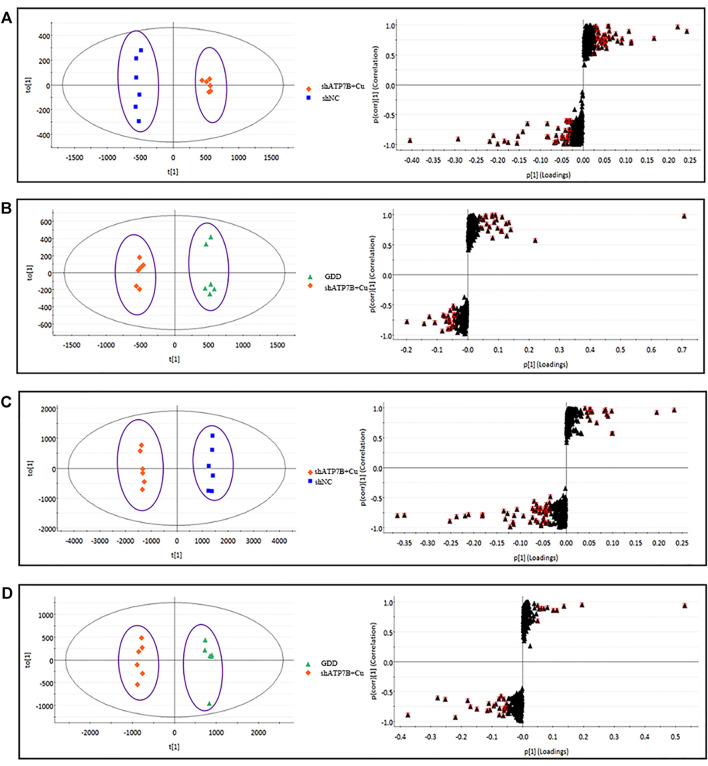
(shNC group, shATP7B + Cu group, and GDD group) OPLS-DA score and its corresponding S-plot for differential analysis between different groups of cell samples in HILIC-Q-TOF/MS negative and positive ESI mode. [**(A)**.NEG-R^2^Y = 1, Q^2^ = 99%; **(B)**. NEG-R^2^Y = 99%, Q^2^ = 98%; **(C)**. POS- R^2^Y = 1, Q^2^ = 98%; **(D)**. POS- R^2^Y = 99%, Q^2^ = 96%].

**FIGURE 7:**
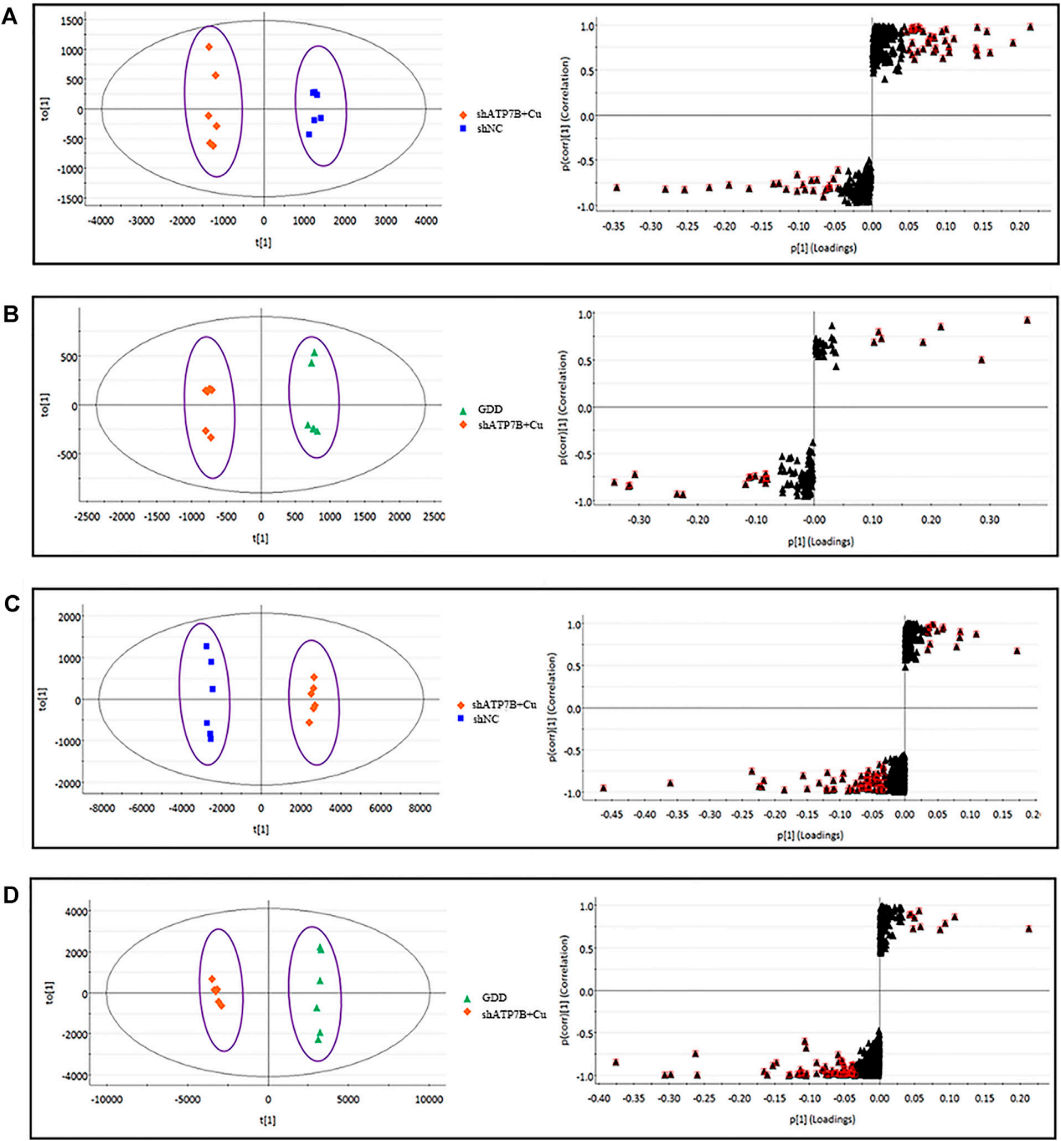
(shNC group, shATP7B + Cu group, and GDD group) OPLS-DA score and its corresponding S-plot for differential analysis between different groups of cell samples in RPLC-Q-TOF/MS negative and positive ESI mode. [**(A)**. NEG-R^2^Y = 99%, Q^2^ = 97%; **(B)**. NEG-R^2^Y = 99%, Q^2^ = 99%; **(C)**. POS- R^2^Y = 1, Q^2^ = 99%; **(D)**. POS- R^2^Y = 1, Q^2^ = 99%].

**FIGURE 8:**
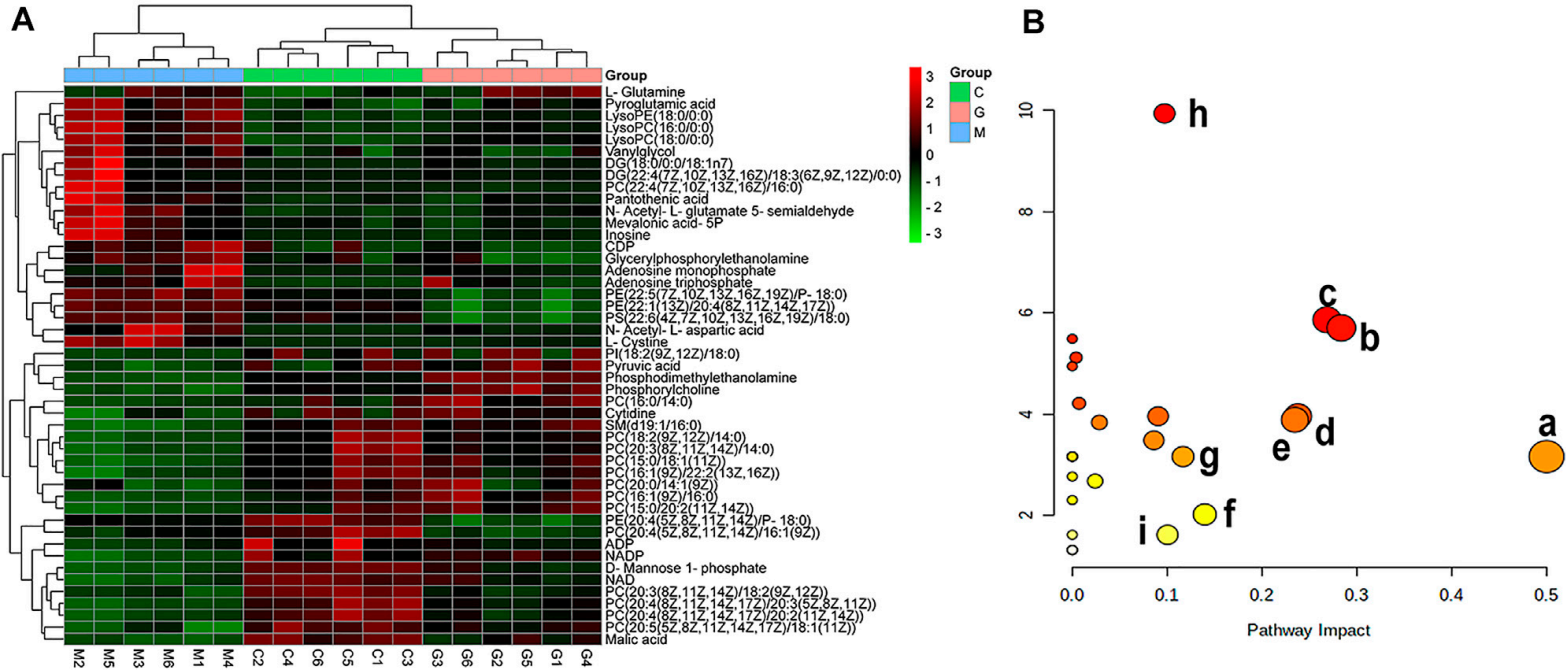
**(A)** Hierarchical clustering heatmap of 47 potential biomarkers. (Columns represent samples in different groups, and rows represent different biomarkers. Different colors, from green to black to red, represent concentration differences, from small to large, in different samples. C, G, and M in figure correspond to shNC group, GDD group, and shATP7B + Cu, respectively); **(B)** Results of metabolic pathway analysis of copper-laden HLD hepatocytes [each point represents a metabolic pathway: (a) D-glutamine and d-glutamate metabolism; (b) alanine, aspartate, and glutamate metabolism; (c) glycerophospholipid metabolism; (d) pyruvate metabolism; (e) nicotinate and nicotinamide metabolism; (f) terpenoid backbone biosynthesis; (g) fructose and mannose metabolism; (h) arginine biosynthesis; (i) Glycolysis/Gluconeogenesis].

For further tracking the specific changes among the three groups, the supervised multivariate OPLS-DA method was used to analyze the variables. The OPLS-DA score map and S-plot diagram in the positive and negative ion modes were obtained, respectively (Figure 6 and Figure 7). The OPLS-DA diagram showed that the shNC and shATP7B + Cu groups were distributed on both sides of t [1] in the OPLS-DA score map, indicating a significant difference in metabolites between the two groups. The S-plot diagram directly showed different metabolites. When the variable is far from the origin, the VIP value is higher, and the contribution to the separation between the two groups is larger. In the figures, the variables with VIP values >1 circled in red boxes are the potential biomarkers between the two groups. The differences in metabolites in all groups were also observed more intuitively using a hierarchical clustering heatmap (Figure 8A). In the figure, the biomarker concentrations in the shATP7B + Cu group significantly differed from those in the shNC group and the GDD group, whereas there was no significant difference in the concentration of biomarkers between the GDD group and the shNC group, indicating that after GDD administration, the cell metabolic pattern reverses to normal or close to normal levels.

### 3.7 Metabolite Identification

The metabolites of each group under the two separate systems of HILIC and C_18_ were statistically analyzed by ANOVA. The metabolites with a VIP value >1 were obtained by selecting the different substances and combining them with the S-plot diagram. Subsequently, the metabolites with a VIP value >1 were screened by HMDB and secondary fragmentation analysis of MS, and the fragment information under high and low collision energy was further matched to verify the chemical structure of the metabolites.

For the metabolites isolated by the HILIC column, the 22 differential metabolites were determined in the shATP7B + Cu group (Table 1), with 13 metabolites increasing and nine metabolites decreasing in concentration. After GDD treatment, levels of pyroglutamic acid, N-acetyl-L-aspartic acid, vanylglycol, L-glutamic acid, glycerylphosphorylethanolamine, pantothenic acid, mevalonic acid-5P, CDP, ATP, N-acetyl-L-glutamate 5-semialdehyde, L-cystine, inosine, malic acid, pyruvic acid, phosphodimethylethanolamine, cytidine, D-mannose 1-phosphate, AMP, ADP, NAD, NADP, and phosphorylcholine (Supplementary Figure S4 and Supplementary Figure S5) were markedly altered in copper-laden HLD hepatocytes. As mentioned earlier, the results of identification under the C_18_ separation system are as shown in Table 2. A total of 25 metabolites related to copper-laden HLD hepatocytes were obtained, with decreased levels of 17 metabolites and increased levels of eight metabolites (Supplementary Figure S6 and Supplementary Figure S7). The results indicated that these metabolites in hepatocytes are the potential biomarkers of the intervention effects of GDD and may be associated with the GDD mechanism of action.

**TABLE 1:** Potential biomarkers in copper-laden HLD hepatocytes detected by HILIC-Q-TOF/MS and their variation tendency (NEG and POS).

No	Features (tR- m/z)	Cal. m/z	Delta m/z (ppm)	VIP	VIP a	Metabolite identification	Formula	Ion form	Fragment ions	Trend	
				C^a^/M^b^	M/G^c^					M/C	G/M
1	1.84_128.0349	128.0353	3.1	7.92	5.16	Pyroglutamic acid	C_5_H_7_NO_3_	M-H	126.7031	↑	↓
2	1.85_133.0132	133.0142	7.5	5.21	2.55	Malic acid	C_4_H_6_O_5_	M-H	115.0065	↓	↑
3	1.09_133.0135	133.0142	5.3	2.81	3.29	Pyruvic acid	C_3_H_4_O_3_	M + FA-H	87.0082	↓	↑
4	5.24_168.0428	168.0431	1.8	1.55	2.13	Phosphodimethylethanolamine	C_4_H_12_NO_4_P	M-H	96.9607, 78.9591	↓	↑
5	1.55_174.0399	174.0408	5.2	4.70	3.33	N-Acetyl-L-aspartic acid	C_6_H_9_NO_5_	M-H	130.0463, 88.0421	↑	↓
6	1.04_183.0665	183.0663	1.1	1.19	1.14	Vanylglycol	C_9_H_12_O_4_	M-H	153.0552, 121.0281	↑	↑
7	1.07_192.0487	192.0508	0	2.00	1.50	L-Glutamic acid	C_5_H_9_NO_4_	M + FA-H	127.9222, 123.9017, 74.0032	↑	↓
8	4.55_214.0485	214.0485	0	1.92	1.90	Glycerylphosphorylethanolamine	C_5_H_14_NO_6_P	M-H	168.9919, 96.9587, 78.9578	↑	↓
9	1.68_218.1020	218.1034	6.4	2.29	1.83	Pantothenic acid	C_9_H_17_NO_5_	M-H	146.0817, 128.0600, 88.0399	↑	↓
10	5.28_242.0803	242.0782	8.7	3.62	3.56	Cytidine	C_9_H_13_N_3_O_5_	M-H	168.0435, 112.9812	↓	↑
11	5.11_259.0226	259.0224	0.8	6.76	2.13	D-Mannose 1-phosphate	C_6_H_13_O_9_P	M-H	229.0061, 96.9607, 78.9578	↓	↑
12	5.35_273.0380	273.0380	0	1.82	1.56	Mevalonic acid-5P	C_6_H_13_O_7_P	M + FA-H	96.9587, 78.9578	↑	↓
13	4.91_346.0555	346.0558	0.9	1.73	1.22	AMP	C_10_H_14_N_5_O_7_P	M-H	134.0459, 96.9667, 78.9578	↑	↓
14	7.19_402.0094	402.0109	3.7	1.56	1.97	CDP	C_9_H_15_N_3_O_11_P_2_	M-H	173.9330, 158.9236, 96.9667	↑	↓
15	8.13_426.0220	426.0220	0	7.58	3.35	ADP	C_10_H_15_N_5_O_10_P_2_	M-H	426.0193, 158.9236	↓	↑
16	9.55_505.9878	505.9885	1.4	2.54	1.23	ATP	C_10_H_16_N_5_O_13_P_3_	M-H	408.0143, 238.8838, 176.9303	↑	↓
17	5.70_662.1057	662.1018	5.9	5.94	2.90	NAD	C_21_H_27_N_7_O_14_P_2_	M-H	425.9982, 78.9596	↓	↑
18	7.91_742.0680	742.0680	0	1.13	1.02	NADP	C_21_H_28_N_7_O_17_P_3_	M-H	96.9687, 78.9578	↓	↑
19	1.01_138.0551	138.0561	7.2	1.23	1.28	N-Acetyl-L-glutamate 5-semialdehyde	C_7_H_11_NO_4_	M + H-2H_2_O	138.0564, 130.0691	↑	↓
20	5.23_184.0754	184.0739	8.1	2.39	4.55	Phosphorylcholine	C_5_H_15_NO_4_P	M + H	124.9771, 98.9858, 84.0804	↓	↑
21	4.60_241.0339	241.0346	2.9	2.13	2.69	L-Cystine	C_6_H_12_N_2_O_4_S_2_	M + H	177.0340, 149.0213, 122.0281	↑	↓
22	2.56_291.0716	291.070	5.5	2.51	1.82	Inosine	C_10_H_12_N_4_O_5_	M + Na	133.0792	↑	↓

a C means shNC, group.

b M means shATP7B + Cu group.

c G means GDD, group.

Change of potential biomarkers in copper-laden HLD, hepatocytes among groups were statistically analyzed by multivariate statistical analysis and ANOVA. Significant changed biomarkers were flagged with (↓) content decreased and (↑) content increased. *p*-values were calculated by Student's t-test (threshold < 0.05).

**TABLE 2:** Potential biomarkers in copper-laden HLD hepatocytes detected by RPLC-Q-TOF/MS and their variation tendency (NEG and POS).

No	Features (t_R_- m/z)	Cal. m/z	Delta m/z (ppm)	VIP	VIP	Metabolite identification	Formula	Ion form	Fragment ions	Trend	
				C^a^/M^b^	M/G^c^					M/C	G/M
1	14.93_750.5472	750.5443	3.9	1.62	1.45	PE [20:4 (5Z,8Z,11Z,14Z)/P-18:0]	C_43_H_78_NO_7_P	M-H	303.2314	↓	↑
2	14.84_776.5684	776.5684	0	2.18	1.51	PE [22:5 (7Z,10Z,13Z,16Z,19Z)/P-18:0]	C_45_H_80_NO_7_P	M-H	462.2978, 331.2635	↑	↓
3	14.55_834.5309	834.5291	2.2	1.03	1.05	PS(22:6 [4Z,7Z,10Z,13Z,16Z,19Z)/18:0]	C_46_H_78_NO_10_P	M-H	327.2201, 283.2606	↑	↓
4	11.30_861.5532	861.5499	3.8	4.17	2.40	PI[18:2 (9Z,12Z)/18:0]	C_45_H_83_O_13_P	M-H	581.3096, 283.2606, 241.0166	↓	↑
5	10.84_866.5963	866.5917	5.3	7.92	5.16	PE [22:1 (13Z)/20:4 (8Z,11Z,14Z,17Z)]	C_5_H_7_NO_3_	M-H	303.2349	↑	↓
6	4.14_482.3258	482.3241	3.5	1.39	1.14	LysoPE (18:0/0:0)	C_23_H_48_NO_7_P	M + H	341.3078, 323.2951	↑	↓
7	3.35_496.3415	496.3398	3.4	2.34	1.81	LysoPC(16:0/0:0)	C_24_H_50_NO_7_P	M + H	184.0744	↑	↓
8	4.03_524.3730	524.3711	3.6	2.16	1.52	LysoPC(18:0/0:0)	C_26_H_54_NO_7_P	M + H	184.0744	↑	↓
9	11.38_605.5479	605.5509	5.0	1.06	1.26	DG (18:0/0:0/18:1n7)	C39H74O_5_	M + H-H_2_O	605.5486, 323.2933	↑	↓
10	11.89_667.5217	667.5272	8.2	1.28	1.18	DG [22:4 (7Z,10Z,13Z,16Z)/18:3 (6Z,9Z,12Z)/0:0]	C_41_H_72_O_5_	M + Na	667.5309, 335.2493	↑	↓
11	11.21_706.5393	706.5381	1.7	2.44	3.58	PC(16:0/14:0)	C_38_H_76_NO_8_P	M + H	184.0744	↓	↑
12	12.60_717.5894	717.5905	1.5	1.86	1.76	SM(d19:1/16:0)	C_40_H_81_N_2_O_6_P	M + H	-	↓	↑
13	9.44_730.5400	730.5381	2.6	2.49	2.19	PC[18:2 (9Z,12Z)/14:0]	C_40_H_76_NO_8_P	M + H	184.0744	↓	↑
14	11.26_732.5525	732.5538	1.8	5.51	6.96	PC[16:1 (9Z)/16:0]	C_40_H_78_NO_8_P	M + H	549.4932, 494.3158, 184.0744	↓	↑
15	12.60_746.5686	746.5694	1.1	3.71	3.31	PC[15:0/18:1 (11Z)]	C_41_H_80_NO_8_P	M + H	184.0744	↓	↑
16	9.91_756.5547	756.5538	1.2	1.91	1.47	PC[20:3 (8Z,11Z,14Z)/14:0]	C_42_H_78_NO_8_P	M + H	363.2894, 184.0744	↓	↑
17	14.08_760.5851	760.5851	0	7.59	6.59	PC[20:0/14:1 (9Z)]	C_42_H_82_NO_8_P	M + H	571.5199, 227.2030, 166.0644	↓	↑
18	12.58_772.5808	772.5851	5.6	2.91	3.54	PC[15:0/20:2 (11Z,14Z)]	C_43_H_82_NO_8_P	M + H	184.0744	↓	↑
19	9.50_780.5561	780.5538	2.9	3.10	1.42	PC[20:4 (5Z,8Z,11Z,14Z)/16:1 (9Z)]	C_44_H_78_NO_8_P	M + H	184.0744	↓	↑
20	9.50_806.5717	806.5694	2.9	2.96	1.97	PC[20:5 (5Z,8Z,11Z,14Z,17Z)/18:1 (11Z)]	C_46_H_80_NO_8_P	M + H	323.2900, 247.2437, 184.0744	↓	↑
21	10.89_808.5849	808.5849	0	5.03	5.33	PC[20:3 (8Z,11Z,14Z)/18:2 (9Z,12Z)]	C_46_H_82_NO_8_P	M + H	623.4977, 321.2749, 184.0744	↓	↑
22	9.94_810.5989	810.6007	2.2	3.39	6.93	PC[22:4 (7Z,10Z,13Z,16Z)/16:0]	C_46_H_84_NO_8_P	M + H	627.5403, 184.0744	↓	↑
23	14.42_812.6144	812.6164	2.5	1.46	1.13	PC[16:1 (9Z)/22:2 (13Z,16Z)]	C_46_H_86_NO_8_P	M + H	627.5352, 184.0744	↓	↑
24	10.18_832.5857	832.5851	0.7	3.85	2.32	PC[20:4 (8Z,11Z,14Z,17Z)/20:3 (5Z,8Z,11Z)]	C_48_H_82_NO_8_P	M + H	184.0744	↓	↑
25	10.82_834.6011	834.6011	0	3.12	1.32	PC[20:4 (8Z,11Z,14Z,17Z)/20:2 (11Z,14Z)]	C_48_H_84_NO_8_P	M + H	184.0744	↓	↑

a C means shNC group.

b M means shATP7B + Cu group.

c G means GDD group.

Change of potential biomarkers in copper-laden HLD, hepatocytes among groups were statistically analyzed by multivariate statistical analysis and ANOVA. Significant changed biomarkers were flagged with (↓) content decreased and (↑) content increased. *p*-values were calculated by Student’s t-test (threshold < 0.05).

### 3.8 Analysis of Metabolic Pathway

The 47 biomarkers were screened using two chromatographic columns mentioned earlier, and their normalized peak area was imported into Metaboanalyst 5.0 software for metabolic pathway enrichment and analysis. The results showed that 30 metabolic pathways were affected in copper-laden HLD hepatocytes (Figure 8B), including glutamate–glutamine metabolism (a); alanine, aspartic acid, and glutamate metabolism (b); glycerol phospholipid metabolism (c); pyruvate metabolism (d); nicotinic acid and nicotinamide metabolism (e); terpene skeleton biosynthesis (f); fructose and mannose metabolism (g); arginine biosynthesis (h); and glycolysis/gluconeogenesis (i). Among these, the glutamate–glutamine metabolic pathway has the most significant influence, with an influence value of 0.5, which is an important metabolic pathway. The KEGG database and the related references were used to further explore the role of differential metabolites.

### 3.9 Effect of Gandou Decoction on Glutamate Level in Copper-Laden Hepatolenticular Degeneration Hepatocytes

**FIGURE 9:**
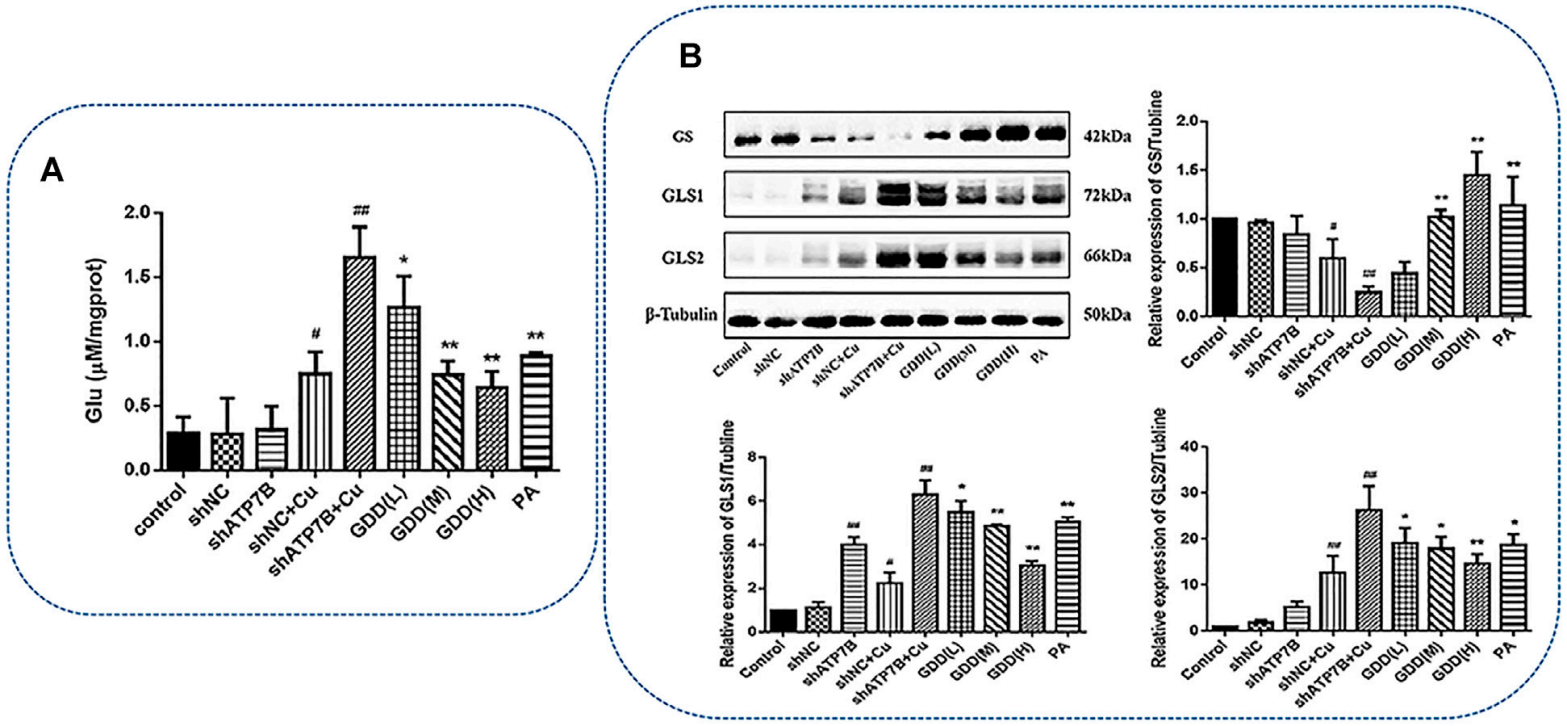
**(A)** Effect of GDD on content of glutamate in copper-laden HLD hepatocytes ( $\overset{�}{x}\pm s,n=3$ ) ^#^ *p* < 0.05, ^##^ *p* < 0.01 compared with control group; ^*^ *p* < 0.05, ^**^ *p* < 0.01 compared with shATP7B + Cu group; **(B)** effects of GDD on GS, GLS1, and GLS2 expression in copper-laden HLD hepatocytes.

Glutamate is an important biomarker of GDD correcting copper-laden HLD hepatocyte metabolic disorder, and therefore, further quantitative analysis of glutamate is necessary for HLD hepatocytes. As shown in Figure 9A, the glutamate content in the shATP7B + Cu group significantly increased (*p* < 0.01). After low-, medium-, and high-dose GDD and PA treatments, the glutamate content in HLD hepatocytes significantly decreased (*p* < 0.05 or *p* < 0.01), suggesting that the glutamate verification results were consistent with the metabolomic results.

In the physiological state, the synthesis, decomposition, uptake, and reabsorption of glutamate is a process of dynamic equilibrium. Glutamine synthetase (GS) and glutaminase (GLS) are critical enzymes in the glutamate biosynthesis pathway. GLS can further be divided into renal-type GLS (GLS1) and liver-type GLS (GLS2). Western blotting experiments were conducted to verify GS, GLS1, and GLS2. The results (Figure 9B) showed that compared with the control group, the shATP7B + Cu group showed a significantly decreased protein expression of GS (*p* < 0.01). In contrast, it was highly expressed in the medium- and high-dose GDD groups. Furthermore, the protein expressions of GLS1 and GLS2 increased significantly in the shATP7B + Cu group and decreased in a dose-dependent manner after intervention with different doses of GDD. All results showed that the corrective effect of GDD on glutamate levels in copper-laden HLD hepatocytes was associated with the upregulation of GS protein expression and downregulation of GLS1 and GLS2 protein expression.

## 4 Discussion

As an effective therapeutic prescription for treating HLD, GDD was reported to have copper removal and hepatoprotective effects against HLD in clinical trials and *in vitro* studies (Hu et al., 2002; Yang and Cheng, 2002; Xue et al., 2007; Li et al., 2013). However, the biological mechanism underlying the basis for these effects remain unknown. In the present investigation, using RNA interference technology, shATP7B RNA was transiently transfected into BRL-3A rat hepatocytes, and a hepatocyte model similar to the pathological HLD was successfully constructed to evaluate the underlying GDD mechanism. BRL-3A cells, which are normal rat hepatogenic cell line cells, are widely used in the study of various liver diseases *in vitro* (Yang et al., 2020), including studies on hepatocyte proliferation, post-hepatectomy regeneration (Chang et al., 2017), hepatotoxicity (Liu et al., 2021), liver function (Hu et al., 2020), oxidative stress (Li et al., 2018), and inflammation (Luo et al., 2021). It was reported that some researchers chose tumor (HepG2 cells) and normal liver cells (BRL-3A cell) to explore the survival time of and cell proliferation in patients with liver cancer (Ye et al., 2019). Another study revealed the regulatory mechanism of acetic acid (AcOH) with regard to liver lipid metabolism in BRL-3A cells (Li et al., 2018). In these studies, the BRL-3A cells were often used to construct a pathological model *in vitro*. Therefore, in our study, an *in vitro* model of HLD was constructed using BRL-3A cells. Subsequently, the HLD cell model was verified *via* RT-PCR and Western blotting, which showed that the levels of ATP7B mRNA and protein in plasmid transfection groups were significantly lower than those in control and shNC groups (Supplementary Figures S1G–L). As the primary pathological organ in HLD, pathological changes in the liver are caused by mutations in the ATP7B gene and copper metabolism disorder. Therefore, 300-μM CuSO_4_ was selected as the optimum modeling concentration for copper-laden HLD hepatocytes through cytotoxicity assays (Figures 1A,B). After this, GDD extract at a concentration of 80 μg/ml showed a strong protective effect on the injury in copper-laden HLD hepatocytes in cell viability evaluation (Figures 1C,D). The study of cell morphology showed that the number of adherent cells significantly increased, and the cell morphology improved in the groups with GDD and PA treatment (Figures 3A–I). In addition, in agreement with the results of PA, GDD could promote the excretion of excessive Cu^2+^ and reduce the accumulation of Cu^2+^ in copper-laden HLD hepatocytes (Figures 2A–J). Thus, GDD may be chelating with Cu^2+^ to reduce the toxicity of Cu^2+^ in hepatocytes and reduces the damage caused by copper-load to HLD hepatocytes.

It is well known that intracellular copper overload can generate the hydroxyl radicals in a Fenton reaction and promote oxidative stress, thereby causing cell injury (Boveris et al., 2012; Valko et al., 2016). Excess-free copper can also promote the generation of ROS in rat liver after acute and chronic exposure to the metal (Jiménez and Speisky, 2000). Intracellular accumulation of copper can also cause an increase in MDA content and LDH release and a decline in GSH/GSSG ratio in the cytosol, further altering mitochondrial morphology and aggravating cell damage (Jiménez et al., 2002; Mladenovic et al., 2014; Musacco-Sebio et al., 2014). SOD activity, MDA content, LDH release, and GSH/GSSG ratio are very important indices, which can reflect the capability of antioxidation (Kumar et al., 2016). The current study revealed that copper exposure significantly enhances ROS production and reduces ROS decomposition in the shATP7B + Cu group; MDA content and LDH release were also significantly increased in this group compared with the control, shNC, and shATP7B groups. In contrast, SOD activity and GSH/GSSG ratio were decreased after increasing copper loads. In addition, GDD could attenuate the oxidative stress induced by copper administration by increasing the SOD activity and GSH/GSSG ratio and decreasing ROS content, MDA content, and LDH release. The results demonstrated that GDD could effectively protect BRL-3A cells from copper-induced hepatic oxidative stress (Figure 4). Further study of the morphological and ultrastructural changes indicated that GDD could reduce injury in copper-laden HLD hepatocytes to varying degrees and has obvious protective effects (Figure 3).

**FIGURE 10:**
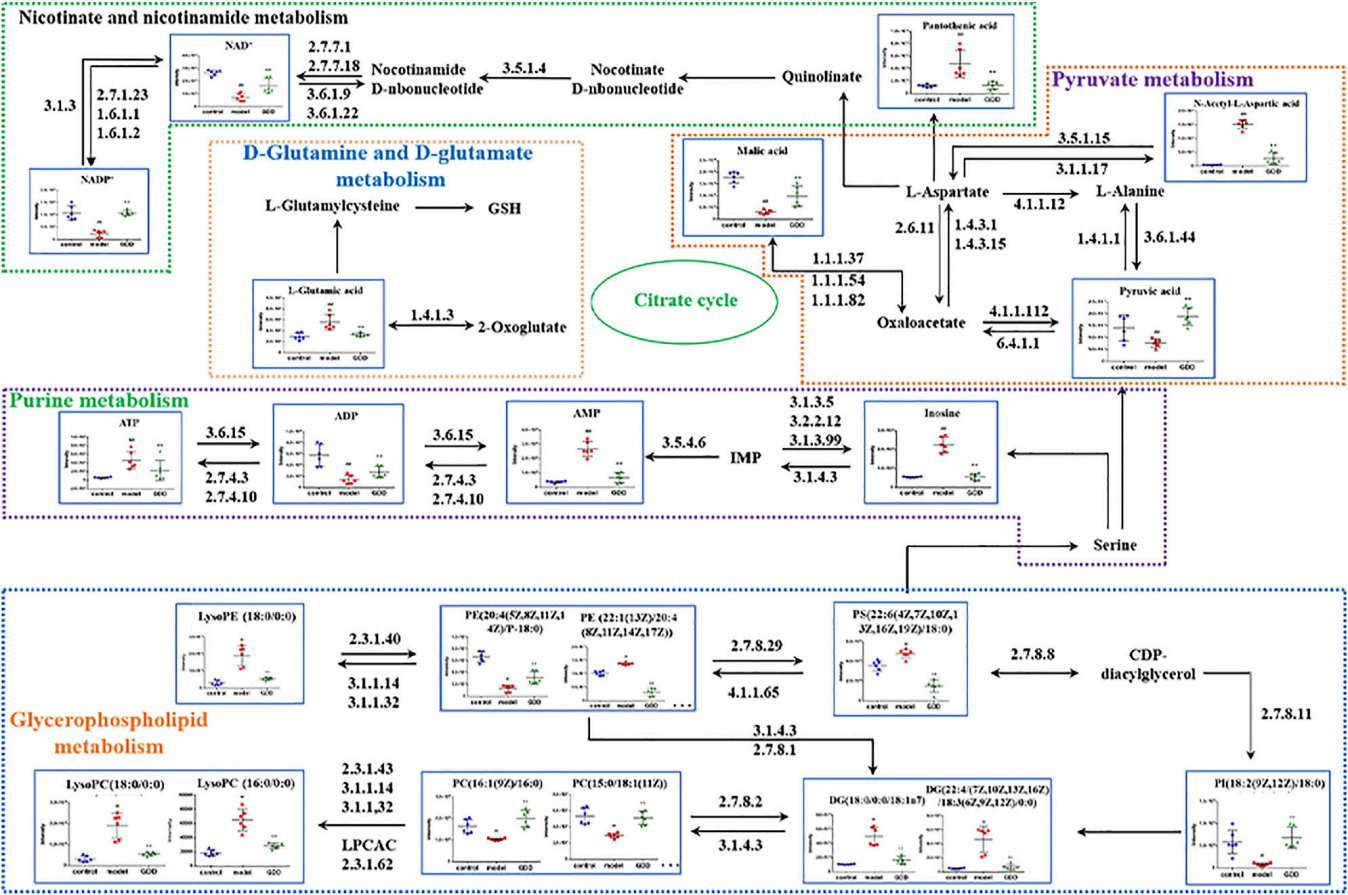
Metabolic network diagram of potential biomarkers in copper-laden HLD hepatocytes by GDD.

To further explore the mechanism of GDD in HLD, cell metabolomics analysis was performed to study the functional metabolites of GDD in the copper-laden HLD hepatocytes. Metabolomic studies found that 47 biomarkers and 30 metabolic pathways are closely linked to the therapeutic effect of GDD on HLD, mainly including d-glutamine and d-glutamate metabolism; alanine, aspartic acid, and glutamic acid metabolism; and glycerophospholipid metabolism. The specific metabolic pathway is shown in Figure 10.

Glutamate is an important amino acid for organisms to maintain a normal physiological function. Glutamate participates in many metabolic processes such as amino acid and sugars metabolism and plays an important role in maintaining nitrogen balance in organisms (Kimball and Jefferson, 2006; Nie et al., 2018). GS and GLS are key enzymes in the glutamate biosynthesis pathway. GS catalyzes glutamate and ammonia to synthesize glutamine in the mammalian liver, which is the main energy source for tumor cells. In mouse and human livers, GS is limited to hepatocytes around hepatic venules. Changes in GS activity are associated with severe hepatic and neurodegenerative diseases, and the absence or malformation of GS can lead to death (Wasfy and Shams Eldeen, 2015; Moreira et al., 2017). GLS exists in mammalian cells in two isozymes, GLS1 (kidney type) and GLS2 (liver type) (Aledo et al., 2000). Some studies have reported that GLS2 is converted to GLS1 in the process of hepatocellular carcinogenesis, and GLS1 was involved in the migration and invasion of hepatocellular carcinoma cells (Zhang et al., 2016). Both GLS1 and GLS2 regulate the antioxidant defense function of cells by increasing the level of GSH and decreasing the level of ROS, protecting cells from oxidative stress. Silencing the expression of GLS1 or inhibiting the activity of GLS1 can disturb this redox homeostasis (Chen et al., 2017; Li et al., 2019) of cancer cells. By evaluating the expression levels of GS, GLS1, and GLS2, the homeostasis of glutamate synthesis and metabolism in the body was measured. Once the homeostasis of glutamate synthesis and metabolism is destroyed, it will cause metabolic disorder, resulting in excessive accumulation of glutamate and consequent toxic effects (McKenna, 2007; Pang et al., 2020). The results showed that the glutamate level in copper-laden HLD hepatocytes was significantly higher than that in the shNC cells. An abnormal increase in glutamate activates the signaling pathway of downstream ROS, causing oxidative damage, and can eventually lead to the death of hepatocytes (Banday and Lejon, 2017). After GDD therapy, glutamate levels in cells tended to be normal, indicating that the protective effect of GDD on HLD may be associated with glutamate metabolism.

Glycerophospholipids are the most abundant phospholipids in organisms. Glycerophospholipids are a major component of the cell and organelle membrane. Furthermore, abnormal metabolism of the glycerophospholipid phosphatidylcholine (PC) is often closely associated with cancer, stroke, and neurodegenerative diseases, and its mechanism is relevant to the increase in stability and integrity of biofilms, reducing the cellular inflammatory response and oxidative stress (Janko et al., 2013). PC, as a substrate for phosphatidylserine (PS) synthesis, participates in acute inflammation (Su et al., 2013). Lysophosphatidylcholine (LPC) is a strong pro-inflammatory regulatory factor, which can stimulate phospholipase A2 through two signaling pathways, and leads to the release of arachidonic acid. Therefore, an irrepressible increase in the level of hemolytic phosphate choline may cause an inflammatory response and then lead to immune damage (Siskind, 2005). Phosphatidylethanolamine (PE) is associated with sustaining the normal structure and function of cells and is closely correlated with the growth, proliferation, and differentiation of cells; it is also an inducer of cellular apoptosis (Zhao and Wang, 2020). Its further hydrolysis will produce lysophatidylethanolamine (LPE) (Vance and Tasseva, 2013).

In this study, the metabolism of glycerophospholipid in copper-laden HLD hepatocytes was significantly altered. The level of intracellular phospholipid choline significantly decreased after CuSO_4_ treatment, whereas the levels of PC, LPE, PE, and PS increased. Changes in these endogenous metabolites suggest that CuSO_4_ can cause a disorder in glycerol phospholipid metabolism, consequently causing liver injury. In the GDD group, these altered metabolites showed varying degrees of callback trend, further indicating that GDD may improve the glycerophosphate metabolism disorder induced by CuSO_4_.

The primary metabolites associated with pyruvate metabolism identified in this study were pyruvate and malate. Pyruvate is the final product of glycolysis and is transported as the main fuel to the mitochondria. It drives ATP production through various biosynthesis pathways, including oxidative phosphorylation and cross-citric acid cycle, and plays an important role in energy metabolism. At the same time, pyruvate can effectively clear free radicals away in the body and play an antioxidant effect (Kao and Fink, 2010). Modern pharmacological studies have shown that pyruvic acid can alleviate organ damage (in organs such as the liver, heart, lung, kidney, and brain) mediated by redox reactions (Gray et al., 2014; Yang et al., 2016). As an intermediate of the tricarboxylic acid cycle, malate is a momentous organic acid produced in the process of cellular metabolism, which improves not only the ability of oxidative phosphorylation and energy metabolism but also the metabolism and antioxidation of liver tissues (Wu et al., 2007; Hikosaka et al., 2021). After CuSO_4_ induction, the contents of pyruvate and malate in the shATP7B + Cu group were decreased compared with the shNC group. It is indicated that CuSO_4_ may destroy the stability of the tricarboxylic acid cycle by affecting intermediate products, causing a disturbance in energy metabolism, and subsequently resulting in damage to hepatocytes. After GDD treatment, pyruvate and malate in cells tended to return to control-like levels, suggesting that the protective effect of GDD on copper-laden HLD hepatocytes may be associated with the regulation of energy metabolism.

In addition, nicotinamide adenine dinucleotide (NAD^+^) and its reduced form (NADH) and nicotinamide adenine dinucleotide phosphate (NADP^+^) and its reduced form (NADPH) are considered to be regulators of glycolysis and mitochondrial oxidative phosphorylation. Studies have shown that increasing NAD^+^ concentrations and increased levels of NAD^+^-dependent enzymes can treat certain metabolic disorders, such as obesity, alcoholic liver disease (Ding et al., 2017; Wang et al., 2018), and even hepatocellular carcinoma (Tummala et al., 2014). Deficiencies or imbalances in cellular NAD(H) and NADP(H) levels perturbed cellular redox state and metabolic homeostasis, leading to redox stress, energy stress, and ultimately diseased states (Ying, 2008; Huang et al., 2018). Compared with the shNC cells, copper-laden HLD hepatocytes showed significantly decreased levels of NAD^+^ and NADP^+^, whereas GDD-treated cells showed an increase in the levels of both metabolites. When cells are under oxidative stress, the AMP/ATP ratio increases (Emerling et al., 2009; Rato et al., 2014). However, studies have also found that during acute inflammation, intracellular ATP levels were abnormally elevated (Bodin and Burnstock, 1998). In this study, the level of ATP in copper-laden HLD hepatocytes increased, whereas the levels of AMP and ADP decreased, which may be because AMP and ADP were converted into ATP under cellular stress. After GDD intervention, the content of related metabolites tended to normal levels. Thus, it can be seen that high copper stimulation can lead to energy metabolism disorders in copper-laden HLD hepatocytes, and GDD may play a protective role by regulating energy metabolism.

In general, we studied the therapeutic mechanism of GDD in copper-laden HLD hepatocytes through cell metabolomics analysis and found 47 biomarkers that can provide new therapeutic targets for the prevention and treatment of HLD. We further provide references in the research of target pathways.

## 5 Conclusion

In this research, a copper-laden HLD hepatocyte model was established based on molecular biology techniques, and the mechanism of GDD in copper-laden HLD hepatocytes was investigated by a combination of pharmacological and cellular metabolomics analyses. The regulatory effect of GDD on glutamate–glutamine metabolism, glycerolipid metabolism, pyruvate metabolism, and other pathways at a metabolic level improved copper-laden HLD hepatocyte metabolism disorders and led to hepatocyte protection. Furthermore, GDD modulated the abnormal biosynthesis of glutamate, an important marker for GDD to correct copper-laden HLD hepatocyte metabolism disorders, by inhibiting GS and activating GLS. These findings provide a new strategy for exploring the protective effect of GDD on copper-laden HLD hepatocytes and provide a new therapeutic target for the prevention and treatment of HLD.

## Data Availability

The original contributions presented in the study are included in the article/Supplementary Material, further inquiries can be directed to the corresponding authors.

## Glossary

AAS: atomic absorption spectrometry
ANOVA: one-way analysis of variance
BPI: Base Peak Intensity
**Cu** ^**2+**^: copper ion
CV: cell viability
**DAPI** 4′: 6-diamidino-2-phenylindole
DCFH-DA: dichlorodihydrofluorescein
GDD: Gandou decoction
GLS: glutaminase
GS: glutamine synthetase
GSH: glutathione
GSSH: oxidized glutathione
HILIC: hydrophilic interaction liquid chromatography
HLD: hepatolenticular degeneration
HRP: horseradish peroxidase
LDH: lactate dehydrogenase
LSD: least significant difference
MDA: Lipid peroxidation
MTT: 3-[4,5dimethyl(thiozol-2-yl)]-2,5-dipheryltetradinmbromide
NC: negative control
OD: optical density
OPLS-DA: orthogonal partial least square discriminant analysis
PA: penicillamine
PAGE: polyacrylamide gel electrophoresis
PCA: principal component analysis
QC: quality control
qPCR: Real-Time PCR
ROS: reactive oxygen species
RP: reversed-phase
SDS: sodium dodecyl sulfate
shRNA: short hairpin RNA
SOD: superoxide dismutase
TCM: traditional Chinese medicine
TEM: transmission electron microscope
UPLC-Q-TOF/MS: Ultra-performance liquid chromatography quadrupole time of flight mass spectrometry
VIP: variable important in the projection
