# Supplementary material for: Protective Mechanism of Gandou Decoction in a Copper-Laden Hepatolenticular Degeneration Model: In Vitro Pharmacology and Cell Metabolomics
Source: Front Pharmacol. 2022 Mar 23;13:848897. doi: 10.3389/fphar.2022.848897 (PMC8984159; doi:10.3389/fphar.2022.848897)
Supplement: Supplementary file 1 [file DataSheet1.pdf]

Supplementary Information

Table of the Contents

1. Supplementary experimental section: Page S1-Page S2
2. Supplementary results and discussion: Page S2-S3
3. Supplementary figures: Figure S1- Figure S7, Page S3-S9
4. Supplementary tables: Table S1-Table S2, Page 10-11.

1. Supplementary Experimental Section

1.1. Establishment and Evaluation of HLD Hepatocyte Model

HLD hepatocyte model was established by RNA interference. The experiment was divided into 6 groups: control group, shNC group, shATP7B-1 group, shATP7B-2 group, shATP7B-3 group and shATP7B-4 group. After washed the 6-well plate with serum-free medium twice, the transfection complex of plasmid DNA-Lipofectamine ™ 2000 was added to the cells. Meanwhile, the serum-free medium without transfection reagent was added to the control group. After incubation for 6 hours, the control medium was replaced and cultured for 48 hours. Follow-up experiments were carried out.

1.1.1. Determination of Transfection Efficiency

After 48 hours, the nucleus was stained with DAPI, and the cell transfection was observed under a confocal laser microscope. The transfection efficiency was the ratio of the number between green fluorescent cells and blue fluorescent cells.

1.1.2. Real-Time PCR (qPCR)

The total RNA of cells in each group was extracted by the Trizol method and then reversely transcribed into cDNA. Then PCR amplification was carried out with the internal reference of β-actin. 10 μL system (including 5 μL of 2×SYBR Green mixture, 1 μL of 10 μM ATP7B upstream primer and downstream primer, 1 μL of cDNA and 2 μL of RNase Free water) was pre-denatured at 95 ℃ for 1 min, then the 40 cycles were operated as follows: denaturation at 95 ℃ for 20 s, annealing at 55 ℃ for 30 s, extension at 72 ℃ for 1 min. Finally, it was extended for 5 min at 72 ℃. Each sample was provided with 3 multiple holes. The experimental results are expressed by the relative expression of the target gene calculated by the $2^{-\triangle\triangle Ct}$ method. PCR primers were synthesized by GenePharma Co. Ltd. (Shanghai, China). All primers were as follows: ATP7B, Forward 5'-CTGGAGAGCTCCATCCTGAG--3', Reverse 5'-CAGGCAGAGAACAACAGACG-3', β-actin, Forward 5'-GAGCGCAAGTACTCTGTGTG-3', Reverse 5'-CCTGCTTGCTGATCCACATC-3'.

1.1.3. Western Blot Assay

The protein isolated from the cell was electrically transferred to a methanol-activated PVDF membrane after separation by 10 % SDS-PAGE. After being blocked by a TBST buffer containing 5 % skim milk for 2 hours, it was incubated overnight at 4 °C with the corresponding first antibody diluent (mouse anti-ATP7B antibody and rabbit anti-β-actin antibody with a dilution ratio of 1: 200). Then, the samples were washed with TBST 3 times, each time for 10 min, and were incubated with HRP labeled goat anti-mouse lgG and HRP labeled goat anti-rabbit lgG (The dilution ratio of them were 1: 10000) for 2 hours. After washed the film, the bands were displayed with an ECL luminescence kit, and each sample was repeated 3 times.

2. Supplementary Results and Discussion

2.1. Evaluation of Hepatolenticular Degeneration Hepatocyte Model

As shown in the figure, there was no green fluorescence expression of GFP in the control group (**Figure S1A**), but the green fluorescence-labeled by GFP could be seen in shNC group and four shATP7B groups (**Figure S1B-F**) after 48 hours transfection. The transfection efficiency of the shATP7B-4 group (**Figure S1F**) was the highest and it was about 70%. RT-PCR results (**Figure S1G-K**) confirmed that compared with the control group, the ATP7B mRNA expression levels of shATP7B-1, shATP7B-2, shATP7B-3, and shATP7B-4 were down-regulated to 35.0% ± 5.1%, 30.5% ± 3.1%, 36.8% ± 4.8% and 29.5% ± 3.9%. Especially in the shATP7B-4 group, the gene interference effect is the strongest. At the same time, western blot results (**Figure S1L**) showed that the expression level of ATP7B protein in the plasmid transfection group was significantly down-regulated ($p < 0.01$), and shATP7B-1, shATP7B-2, shATP7B-3 and shATP7B-4 were down-regulated to 37.2% ± 3.4%, 32.6% ± 5.1%, 34.3% ± 6.0% and 30.5% ± 2.8% respectively. In summary, the shATP7B-4 group has the strongest transfection effect and can be used for the establishment of copper-loaded HLD hepatocyte model.

2.2. The Method Validation of Cell Metabolomics

Under two separation systems, a characteristic ion peak (Rt-m/z HILIC column: negative ion 3.99 min-237.0911, positive ion 4.55 min-754.1806, C_{18} column: negative ion 2.68 min-391.2828, positive ion 2.92 min-532.3852) was randomly selected from each QC sample in dual ESI mode for UPLC-MS methodology verification (**Table S2**). The instrument precision results of Q-TOF/MS by hydrophilic column separation showed that the relative standard deviations (RSD) of retention time and peak area were below 0.16% and 6.81%, respectively. Similarly, the RSD results of the reverse chromatographic separation system were inferior to 0.35% and 5.05%. In addition, the sample stability results showed in the HILIC system that RSD of retention time and peak area were under 0.24% and 7.97%, and in the C_{18} system that were under 0.31% and 6.65%. The overlapping spectral analysis of QC samples proved that the instrument

was stable and the metabolomics analysis method was reliable.

3. Supplementary Figures

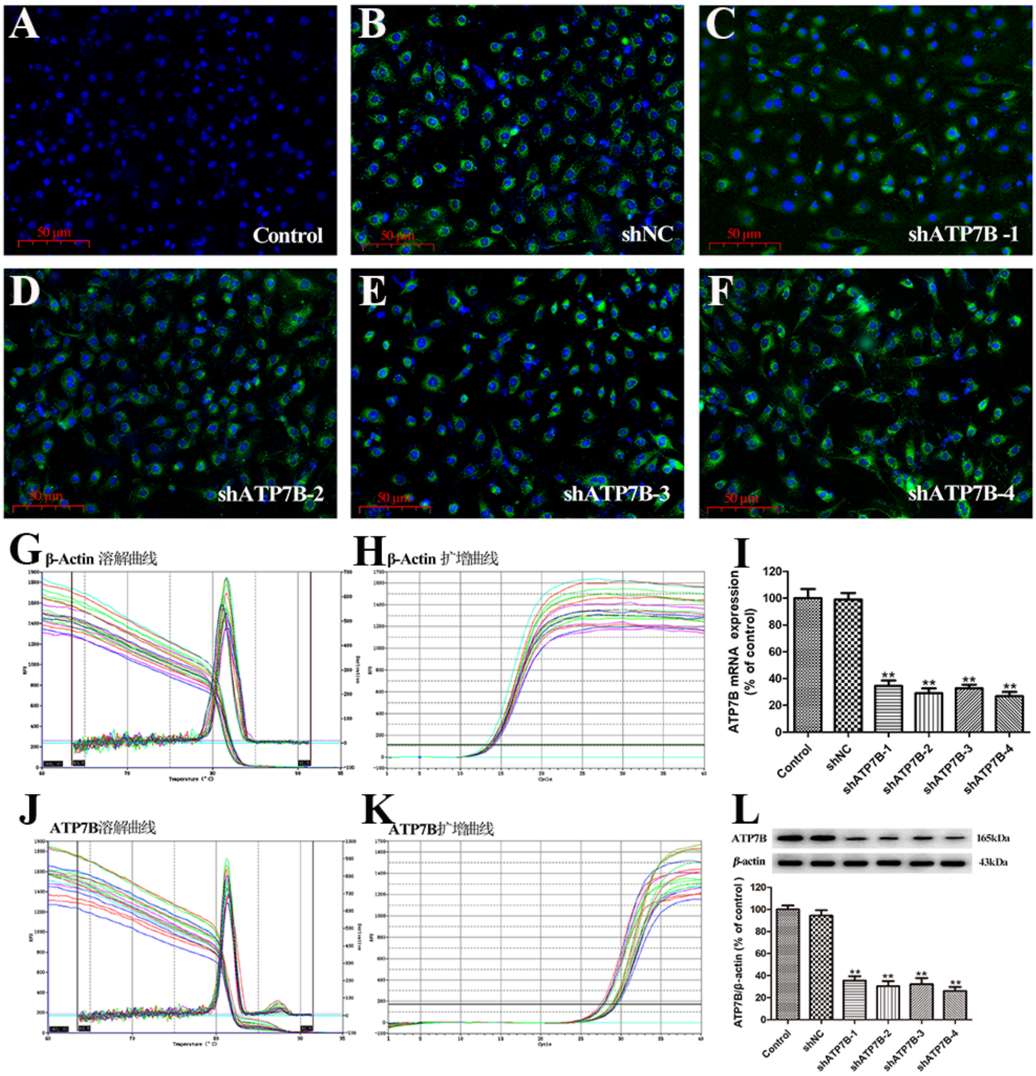

Figure S1. (A-F) Fluorescence expression of BRL-3A cells after 48 hours of shRNA transfection under fluorescence microscope (magnification = 200 ×, 50 μm); (G-H, J-K) Melt curve and amplification Plot of β-Actin and ATP7B; (I) Detection of ATP7B mRNA expression in BRL-3A cells after transfection by RT-PCR ($\bar{x} \pm s, n = 3$), $^{**}p < 0.01$ compared with control group; (L) Detection of ATP7B protein expression in BRL-3A cells after transfection by Western blot ($\bar{x} \pm s, n = 3$), $^{**}p < 0.01$ compared with control group.

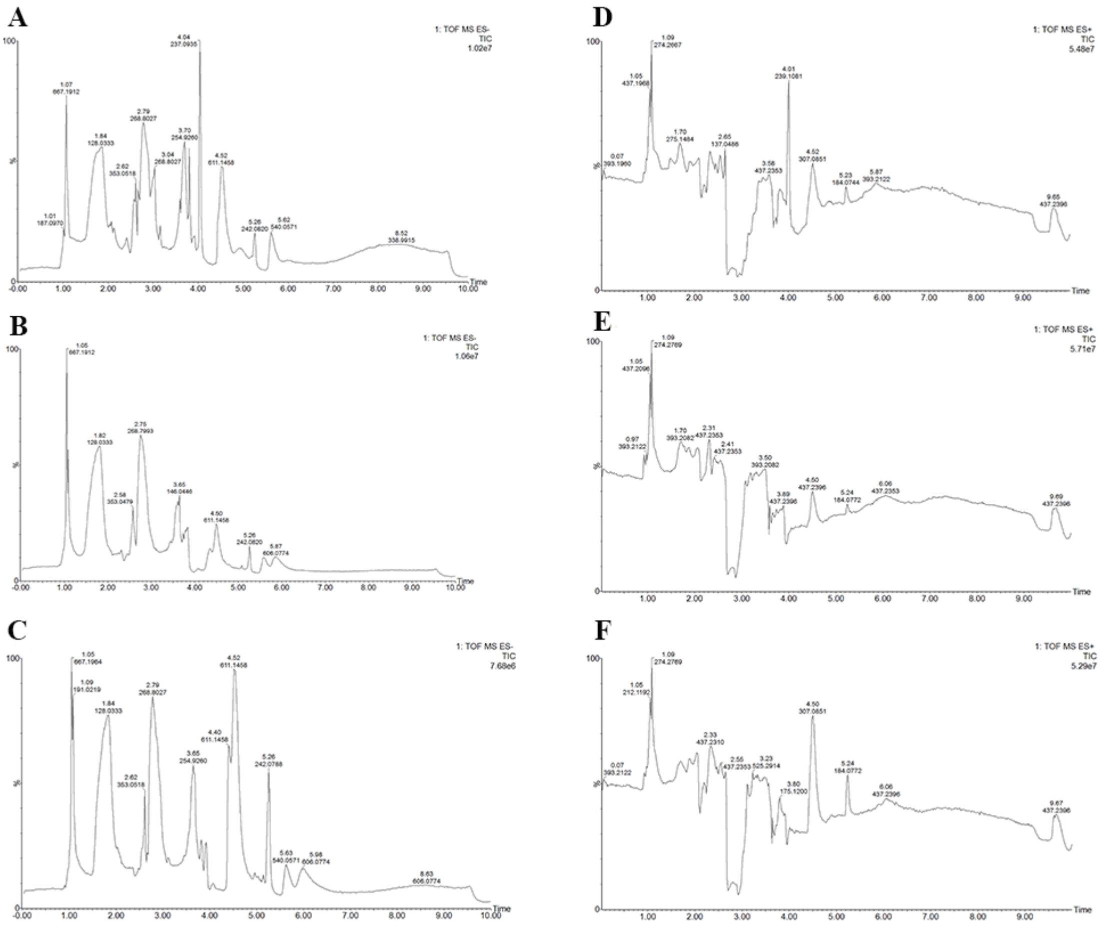

Figure S2. The TIC profile of HLD hepatocyte samples by HILIC-Q-TOF/MS. (A-C) Negative ESI mode. (D-F) Positive ESI mode. (A, D) shNC group. (B, E) shATP7B+Cu group. (C, F) GDD group.

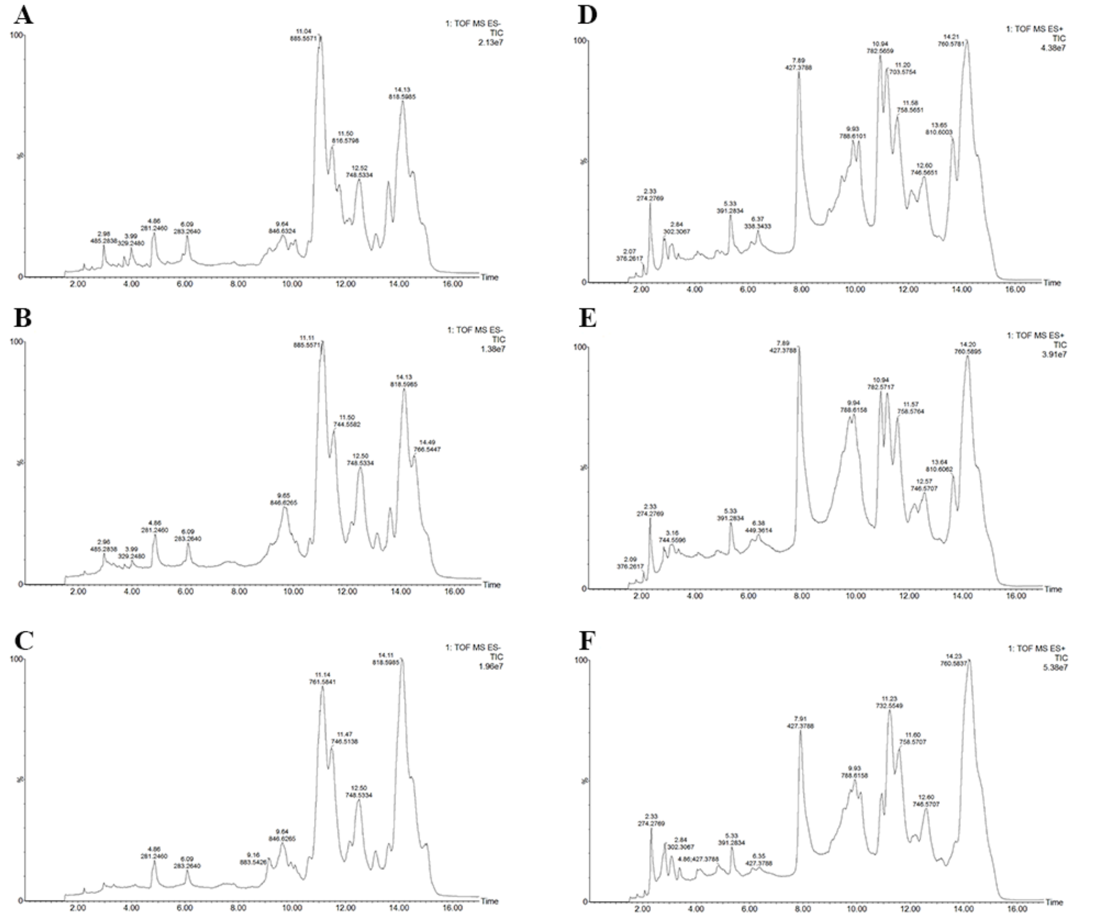

Figure S3. The TIC profile of HLD hepatocyte samples by RPLC-Q-TOF/MS. (A-C) Negative ESI mode. (D-F) Positive ESI mode. (A, D) shNC group. (B, E) shATP7B+Cu group. (C, F) GDD group.

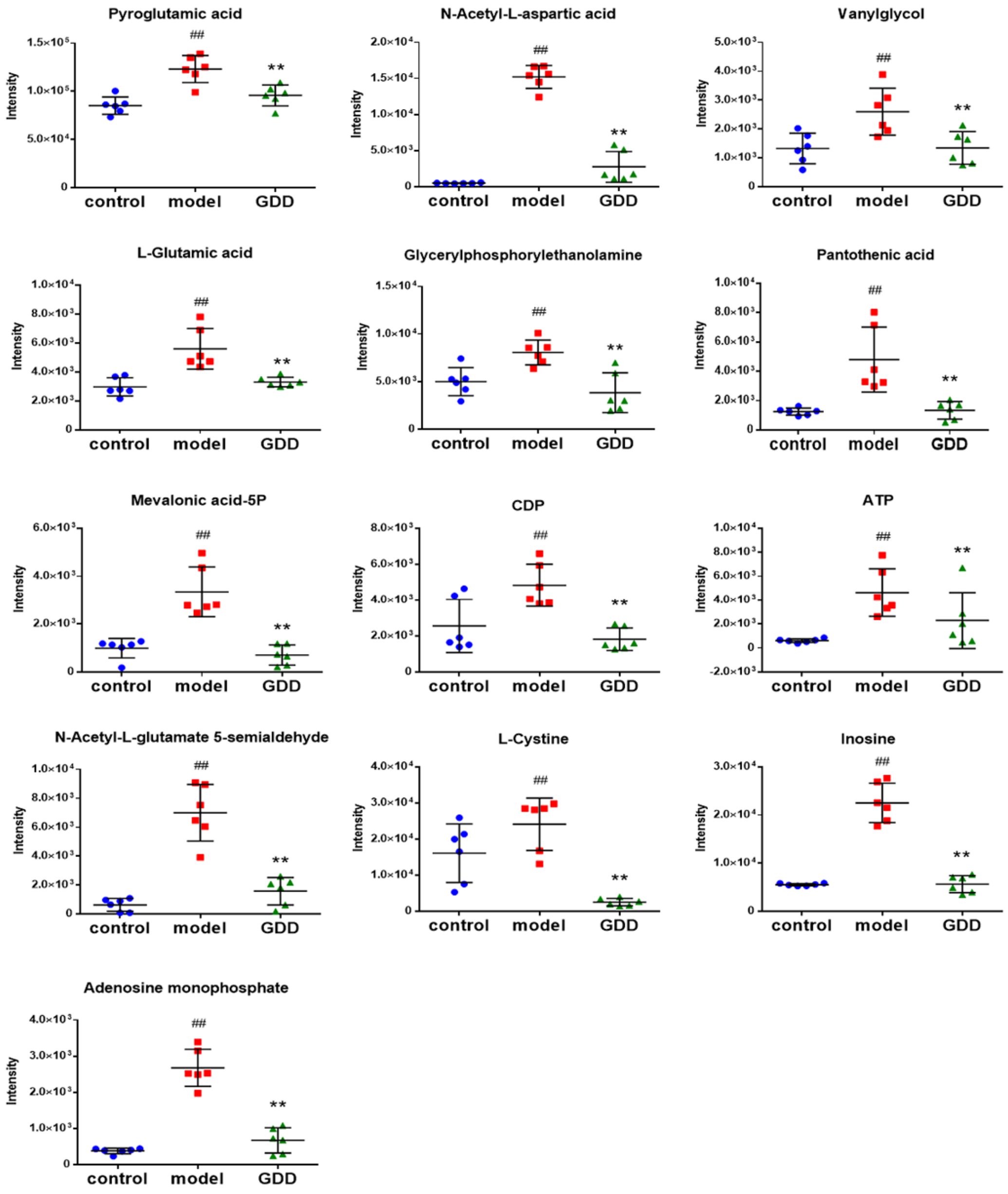

Figure S4. HILIC-Q-TOF/MS detected the relative content of 13 differential metabolites down-regulated by GDD. (In the figure, control, model and GDD represent shNC group and shATP7B+Cu group and GDD group in turn; $^{\#\#}p$ < 0.01 compared with control group; $^{*}p$ < 0.05, $^{**}p$ < 0.01 compared with shATP7B+Cu group; Mean ± SD, n = 6).

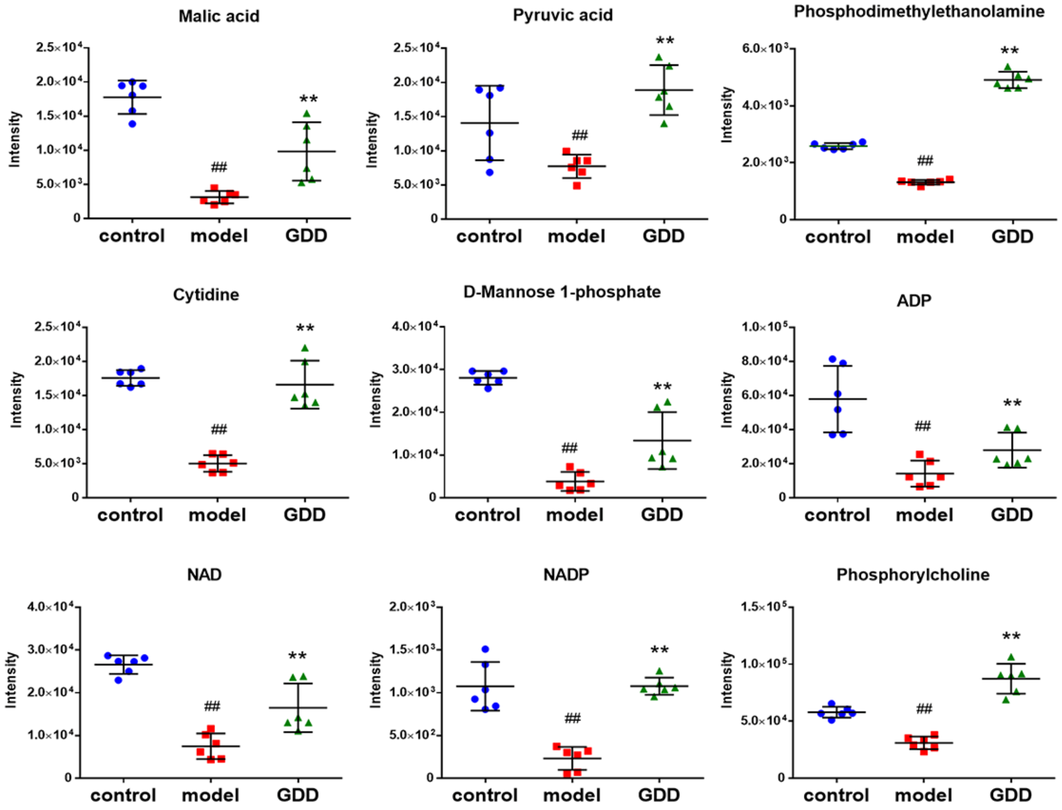

Figure S5. HILIC-Q-TOF/MS detected the relative content of 9 differential metabolites up-regulated by GDD. (In the figure, control, model and GDD represent shNC group and shATP7B+Cu group and GDD group in turn; ##$p < 0.01$ compared with control group; **$p < 0.01$ compared with shATP7B+Cu group; Mean ± SD, n = 6).

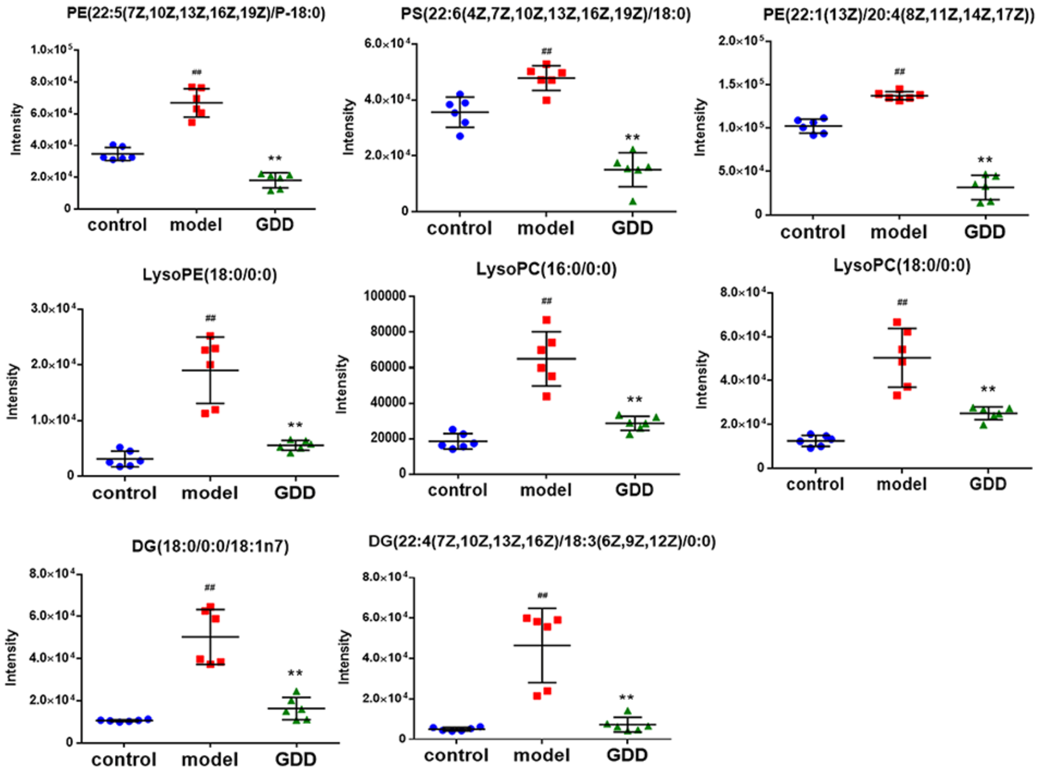

Figure S6. RPLC-Q-TOF/MS detected the relative content of 8 differential metabolites down-regulated by GDD. (In the figure, control, model and GDD represent shNC group and shATP7B+Cu group and GDD group in turn; ##$p < 0.01$ compared with control group; **$p < 0.01$ compared with shATP7B+Cu group; Mean ± SD, n = 6).

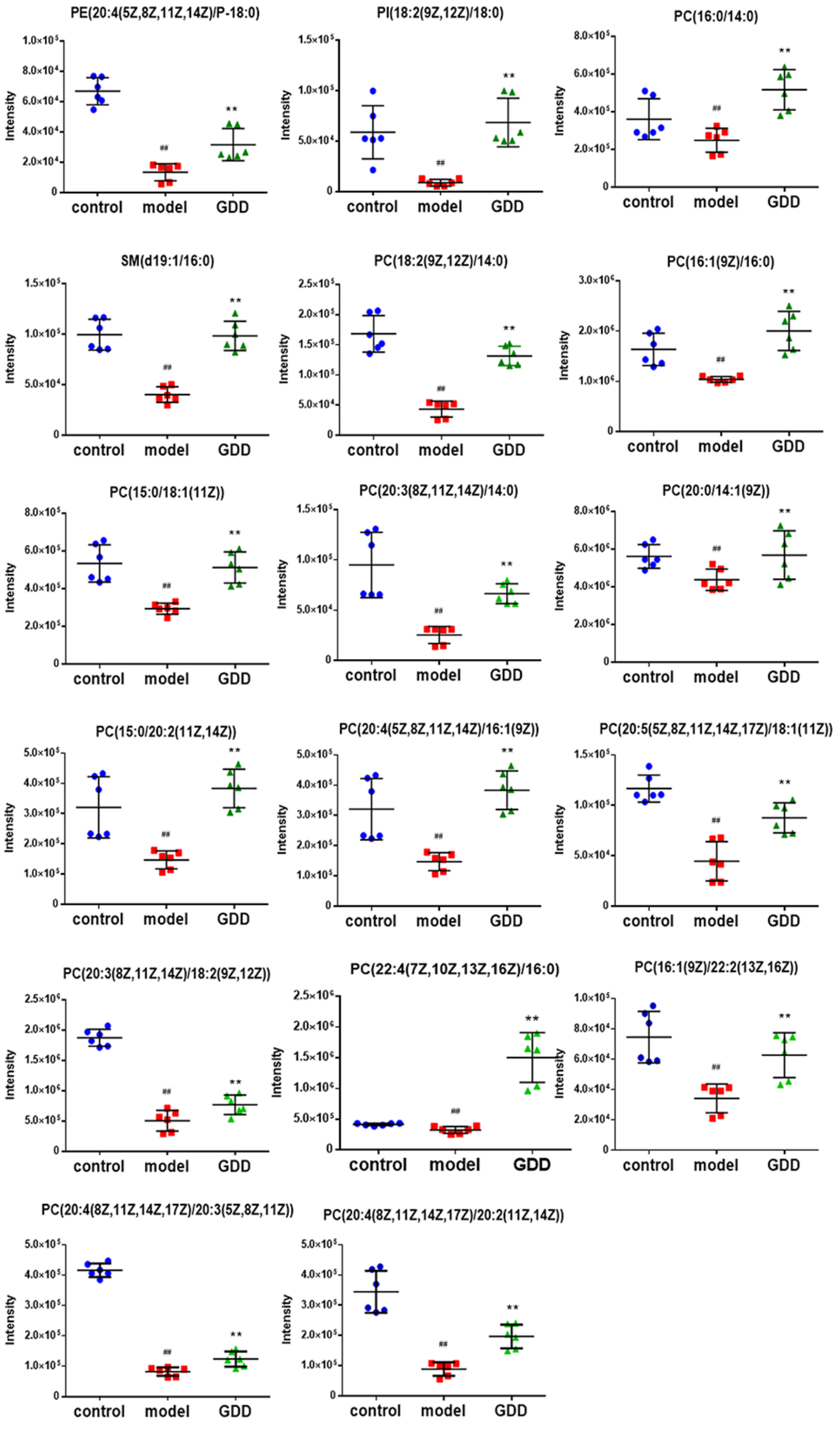

Figure S7. RPLC-Q-TOF/MS detected the relative content of 17 differential metabolites down-regulated by GDD. (In the figure, control, model and GDD represent shNC group and shATP7B+Cu group and GDD group in turn; $^{##}p < 0.01$ compared with control group; $^{**}p < 0.01$ compared with shATP7B+Cu group; Mean ± SD, n = 6).

4. Supplementary table

Table S1. Detailed information of the crude drugs composed in GDD

Latin name	Chinese name	Place of Origin	Supplier	Bach number	Voucher Specimens Reserve	Voucher number
**Rheum palmatum L.**	Da huang	Gansu, China	Beijing Tongrentang Pharmacy Co., Ltd.	20180106	Anhui University of Chinese Medicine, Hefei, China	18056
**Coptis chinensis Franch.**	Huang lian	Sichuan, China	Beijing Tongrentang Pharmacy Co., Ltd.	20180101	Anhui University of Chinese Medicine, Hefei, China	18031
**Curcuma longa L.**	Jiang huang	Sichuan, China	Beijing Tongrentang Pharmacy Co., Ltd.	220180120	Anhui University of Chinese Medicine, Hefei, China	18059
**Lysimachia christinae Hance**	Jin qian cao	Sichuan, China	Beijing Tongrentang Pharmacy Co., Ltd.	20180113	Anhui University of Chinese Medicine, Hefei, China	18070
**Alisma orientale (Sam.) Juzep.**	Ze xie	Sichuan, China	Beijing Tongrentang Pharmacy Co., Ltd.	20180107	Anhui University of Chinese Medicine, Hefei, China	18052
**Panax notoginseng (Burk.) F. H. Chen**	San qi	Yunnan, China	Beijing Tongrentang Pharmacy Co., Ltd.	20180109	Anhui University of Chinese Medicine, Hefei, China	18067

Table S1. Methodological results under HILIC and C_{18} separation systems

Ion mode	HILIC-NEG（RSD-%）		HILIC-POS（RSD-%）		C18-NEG（RSD-%）		C18-POS（RSD-%）	
metabolite	3.99 min-237.0911		4.55 min-754.1806		2.68 min-391.2828		2.9 min-532.3852	
	Time	Area	Time	Area	Time	Area	Time	Area
Instrument precision	0.13	6.18	0.16	5.96	0.35	5.05	0.21	3.72
Sample stability	0.16	7.97	0.24	5.54	0.31	6.65	0.22	4.96
